# Between Leaves, Prayers, and Healing Hands: A Cross-Regional Scoping Review of Ritual Healers and Plant-Based Community Knowledge

**DOI:** 10.3390/plants15162467

**Published:** 2026-08-14

**Authors:** Elaine de Lima de Jesus, Maria Fernanda Barros de Oliveira Brandão, Luana Luzia Santos Pires, Erica Letícia Gomes Alves, Anne Júlia Sousa Silva, Ana Claudia da Silva Bahia, Fernanda Lula Figueiredo Cambuí, João Victor Quintas dos Santos, Davyson de Lima Moreira, Ygor Jessé Ramos

**Affiliations:** 1 Farmácia da Terra Laboratory, Faculty of Pharmacy, Federal University of Bahia, Rua Barão de Jeremoabo, 147, Ondina, Salvador 40170-115, BA, Brazil; elainejesus.farmacia@gmail.com (E.d.L.d.J.); maria.barros@ufba.br (M.F.B.d.O.B.); llspires@yahoo.com.br (L.L.S.P.); ericaleticia2008@gmail.com (E.L.G.A.); juliasilvaxx@hotmail.com (A.J.S.S.); claudia.bahia@ufba.br (A.C.d.S.B.); fernanda.lula@ufba.br (F.L.F.C.); joao.quintas@ufba.br (J.V.Q.d.S.); 2 Graduate Program in Plant Biology, Institute of Biology, Rio de Janeiro State University, Maracanã, Rio de Janeiro 20550-013, RJ, Brazil; davysonmoreira@jbrj.gov.br; 3 Research Institute of the Rio de Janeiro Botanical Garden, Rua Pacheco Leão 915, Jardim Botânico, Rio de Janeiro 22460-030, RJ, Brazil

**Keywords:** ethnobotany, medicinal plants, traditional knowledge, ritual healing, medical pluralism

## Abstract

Plant-based healing practices integrate botanical resources, ritual speech, gesture, faith, spiritual mediation, protection, and social recognition within complex community systems of care. This cross-regional scoping review mapped the traceable literature on plant-based knowledge associated with benzedeiras, benzedores, rezadeiras, rezadores, and functionally analogous specialists. The formal scoping-review corpus comprised 54 records retrieved through structured database searches. A separate contextual layer of 132 supplementary records, identified through citation tracking and targeted searches using emic specialist terms, broadened interpretation but was not treated as methodologically equivalent to the PRISMA-derived corpus. Across the 186 analytical records, 74 harmonized specialist terms and 212 botanical entries associated with therapeutic, ritual, spiritual, or community-based practices were identified. The botanical matrix comprised 68 families and 166 genera, with Lamiaceae, Asteraceae, Solanaceae, and Fabaceae as the most represented families. The results reveal terminological diversity, regional asymmetries in documentation, and the predominance of broad labels alongside terms with greater emic density. Plants operate as pharmacological resources, ritual supports, spiritual mediators, instruments of protection, territorial markers, and performative elements of healing. These specialists should be understood as mediators of complex therapeutic systems. The findings represent the indexed and traceable supplementary literature analyzed and should not be interpreted as a comprehensive inventory of ritual healing traditions worldwide.

## 1. Introduction

Plant-based healing practices constitute one of the most persistent forms of community health knowledge, particularly in rural, Indigenous, Afro-descendant, peasant, peri-urban, and traditional societies. In these contexts, care is not organized solely around the empirical identification of medicinal species, plant parts, preparation methods, and therapeutic indications but also involves a complex network of broader relationships that encompass the body, environment, territory, spirituality, social memory, and community recognition in everyday life [1,2,3]. From an ethnobotanical perspective, this integration requires attention not only to the symbolic position of plants but also to their taxonomic identity, vernacular names, parts used, forms of preparation, routes of administration, specific applications, and the specialists responsible for selecting, prescribing, or ritually mobilizing them. Thus, the medicinal plant should not be understood exclusively as a pharmacological resource, but rather as an element integrated into local systems for interpreting and diagnosing illness, providing protection, alleviating suffering, and restoring individual and collective balance [4,5,6].

In many contexts, leaves, roots, barks, seeds, resins, oils, smoke, baths, and infusions simultaneously function as remedies, ritual supports, markers of belonging, diagnostic instruments, means of cleansing, offerings, or vehicles of spiritual mediation. In such cases, therapeutic efficacy is constructed through the articulation of biochemical, sensory, symbolic, performative, and social dimensions, making it insufficient to rigidly separate “medicine”, “religion”, and “ritual” as independent domains [7,8,9,10].

This symbolically charged use is often mediated by ritual healing specialists linked to traditional and community-based systems of care, whose therapeutic authority variably involves plant knowledge, verbal practices, gestures, spiritual mediation, symbolic diagnosis, protection, cleansing, counseling, and social recognition. The authority of these ritual healing specialists does not derive solely from technical mastery of plant species but also involves reputation, moral conduct, lineage, experience, gift, secrecy, perceived efficacy, territorial belonging, mediation with entities or divinities, and community trust. Accordingly, emic categories such as benzedeira, rezador, curandero, sangoma, nganga, yachak, machi, tohunga, or ngangkari condense local conceptions of personhood, body, illness, vulnerability, spirituality, and therapeutic legitimacy that, although potentially comparable in certain functional aspects, originate from distinct territories and cultural systems. Therefore, the indiscriminate translation of these terms as “traditional healer”, “folk healer”, or “spiritual healer” may facilitate bibliographic indexing but often reduces the emic and epistemological density of these social positions [11,12].

Based on this premise, the analytical category adopted in this study includes terms such as benzedeiras and benzedores, rezadeiras and rezadores, curanderos and curanderas, sangomas, ngangas, yachak/yachakkuna, kahuna, ngangkari, cunning folk, and other culturally situated designations. This category does not assume full equivalence among these figures but allows the examination of functional approximations among distinct practices while preserving the historical, linguistic, religious, and territorial specificity of each healing system [8,12].

In contemporary societies, these practices are also articulated with medical pluralism. Individuals and communities frequently move among primary health care units, hospitals, pharmacies, churches, terreiros, plant markets, domestic spaces, and traditional specialists according to perceived severity, cost, accessibility, trust, previous experience, and cultural interpretations of illness. Such movement should not necessarily be read as a therapeutic contradiction but rather as a pragmatic care strategy in which biomedicine, medicinal plants, prayer, spiritual protection, and community support may coexist within complex therapeutic itineraries [11].

Within these articulations of praxis lies benzimento. Operationally, the term “benzimento” is understood as a ritual practice of care in which prayer, gesture, faith, secrecy, community recognition, and, in many cases, material elements such as leaves, branches, water, oil, smoke, salt, or bodily signs are articulated to protect, cleanse, relieve, heal ailments, restore balance, and embrace different forms of suffering. In this review, benzimento is retained as a situated Brazilian comparative case rather than as a predefined metric for classifying other traditions. Cross-cultural comparison was first organized through independently coded analytical domains: community recognition, plant use, ritualized speech, bodily gesture, material support, spiritual mediation, protection or cleansing, diagnosis, and therapeutic or restorative purpose. Benzimento was considered only after this domain-based coding, as one configuration among several documented healing systems. Within this comparative framework, religious and ritual dimensions reveal the contexts that organize the selection, preparation, application, and meaning of plants within culturally situated healing practices.

The transmission of these forms of knowledge occurs through diverse pathways, including family learning, everyday observation, initiation, spiritual calling, personal experiences of illness, dreams, revelations, community participation, and prolonged coexistence with recognized practitioners [13,14,15,16,17,18,19,20]. This continuity, however, has been challenged by processes of urbanization, migration, schooling, religious intolerance, biomedicalization, habitat loss, aging of specialists, and declining interest among younger generations. At the same time, the digital circulation of knowledge previously restricted to specific community contexts produces new forms of visibility, appropriation, patrimonialization, and disputes over authority [5,6].

Thus, three main gaps guide this review. The first is terminological and epistemological: the international literature uses broad indexing categories, such as “traditional healer”, “healer”, “folk healer”, or “shaman”, which facilitate bibliographic retrieval but may obscure differences among blessing specialists, prayer healers, herbalists, priests, spiritual mediators, raizeiros, shamans, and therapists from codified medical systems. The second gap is analytical: ethnobotanical studies tend to privilege lists of species, plant parts used, and therapeutic indications, whereas anthropological studies describe prayers, gestures, initiations, secrets, spiritual diagnoses, and community authority; however, in ritual contexts, plants and symbolic practices constitute inseparable dimensions of care. Nevertheless, the integration of these dimensions also depends on making the botanical evidence explicit, including the plant taxa involved, their documented uses, the specialists associated with them, and the geographical and cultural contexts in which these relationships were reported. The third gap is sociopolitical and intergenerational: the relationships among knowledge transmission, community legitimacy, gender, religiosity, medical pluralism, public health, coloniality, religious racism, systemic legal restrictions, institutional exclusion, and the recognition of traditional knowledge remain insufficiently systematized.

In light of these gaps, Brazilian benzimento offers a particularly relevant situated case, as it articulates ritual speech, gesture, faith, secrecy, plant-based support, protection, cleansing, and community recognition within widely distributed yet locally differentiated care practices. By including it as one comparative configuration, this review does not seek to universalize benzimento or transform heterogeneous practices into variants of a single tradition. Rather, benzimento is discussed after independent domain-based coding, while the cultural, linguistic, religious, and territorial specificities of specialists documented across different regions are preserved.

Therefore, this cross-regional scoping review, combining narrative synthesis and descriptive-exploratory analysis, aims to map and interpret how the traceable scientific literature describes ritual healing specialists and plant-based community knowledge, using a domain-based comparative framework and discussing Brazilian benzimento as a situated case rather than as a universal model. Specifically, it seeks to identify emic terms and harmonized categories of ritual healing specialists; compare independently coded domains of plant use, speech, gesture, material support, protection, spiritual mediation, diagnosis, and community authority; synthesize the botanical repertoires associated with these practices, including plant taxa, botanical families, specific documented uses, preparations, specialist associations, and geographical contexts; and discuss transmission, community legitimacy, legal and institutional vulnerability, and interfaces with medical pluralism and public health.

## 2. Results and Discussion

### 2.1. Cross-Regional Mapping of Ritual Healing Specialists and Terminological Diversity

The formal corpus derived from the structured PRISMA-ScR pathway comprised 54 records. A separate contextual evidence layer contained 132 supplementary records identified through citation tracking and targeted searches using emic terms, yielding 186 analytical records in total and 74 harmonized specialist terms. Because the two layers were methodologically distinct, only the 54 records were treated as the PRISMA-derived scoping-review corpus; the 132 supplementary records supported contextual, terminological, and comparative interpretation. The continental allocation of the 186 records is reported as a description of corpus composition in Supplementary Figure S11: Asia, 60 records; South America, 44; Africa, 30; Europe, 25; North America, 12; Oceania, 12; and transregional records, 3.

The observed distribution is partly determined by the terminology through which studies are indexed and translated. The broad category “traditional healer” accounted for 46 of 186 analytical records (24.7%), followed by “healer”, “benzedor(a)/rezador(a)”, “yachak/yachakkuna”, and “curandero/curandera” (Supplementary Table S3). This frequency describes the labels assigned in the reviewed literature, not the cultural relevance of the corresponding specialists. More situated terms, such as benzedeira/benzedor, rezadeira/rezador, sangoma, inyanga, hakim, vaidya, albularyo, tohunga, and rongoā healer, preserve greater emic specificity when accompanied by ethnographic description because they designate locally defined forms of therapeutic authority and intervention.

The central interpretation is that terminological diversity constitutes a substantive result of the review. Specialist categories are not equivalent to one another; rather, they condense characteristic regimes of authority, transmission, cosmology, diagnosis, relationships with plants, spiritual mediation, and community legitimation. Therefore, cross-cultural comparison was conducted through independently coded analytical domains and controlled comparison of documented practices, rather than through the automatic translation of different healing agents into a universal category of “traditional healer” or their classification against a predefined benzimento profile [11,12].

Specialist records and botanical entries are different units and are no longer juxtaposed in the main text. A specialist record identifies a source that described a healing specialist, whereas a botanical entry requires an extractable plant name or ethnospecies linked to that source. The difference between the two counts therefore measures botanical reporting and extraction yield within the included literature. Plant-rich ethnobotanical surveys contribute many botanical entries, while anthropological or historical studies may describe specialists in depth without supplying taxonomic lists. The transferred display in Supplementary Figure S11 is retained solely to document this difference in source structure.

### 2.2. The Position of Plants in Ritual Healing Systems

Plants assume different positions in ritual healing systems, as summarized in Table 1 (Tables S1 and S5–S7). In some contexts, they constitute the main therapeutic element of the practice, especially when the specialist’s authority is anchored in knowledge of species, plant parts used, preparation methods, and forms of administration. In others, they operate as ritual supports, in which leaves and branches are combined with other elements to prepare baths, waters, oils, or smoke, and are activated by words, gestures, touch, blowing, or blessings. They may also act as cosmological mediators, articulating relationships among humans, spirits, ancestors, divinities, territory, dreams, visions, and protection, or remain as peripheral records when botanical identification or ritual description is insufficient [21,22,23,24].

A total of 212 records distributed across 68 botanical families were found, with taxonomic standardization supported by sources such as POWO/WCVP whenever possible. These records include species identified at the species level, genus- or family-level records, taxa indicated with nomenclatural uncertainty, and plants not identified at the species level. Therefore, the data should be interpreted as documentary plant records or plant-use units. South America concentrated 102 continental attributions, followed by Asia, with 79; Africa, with 33; Oceania, with 5; Europe, with 4; and transregional records, with 2 (Table 2; Supplementary Table S14). This distribution differs from the count of specialists: Asia showed the highest number of specialist records, whereas South America concentrated greater botanical density, mainly due to Brazilian and Andean studies with more detailed plant lists, such as Bastos et al. [23], Sousa et al. [25], Armijos et al. [24], and Armijos et al. [26].

Lamiaceae, Asteraceae, Solanaceae, and Fabaceae were the most frequent families. Lamiaceae and Asteraceae are expected in ethnobotanical corpora because of the broad presence of aromatic, digestive, anti-inflammatory, topical, food, and ritual species, in addition to being among the families most frequently reported in surveys of medicinal plants and traditional uses [27,28,29]. Solanaceae occupies a singular position because it includes food plants, medicinal plants, and species with high psychoactive or ritual potency, such as *Brugmansia*, *Datura*, and *Nicotiana*, requiring joint interpretation of therapeutic use, ceremonial context, dose, route of administration, and biological risk [30,31]. Fabaceae appears as a family of high cultural plasticity, including arboreal, resinous, food, anti-inflammatory, and protective species recorded in local systems of traditional medicine and ethnobotany [32,33,34]. The presence of “unidentified” categories reinforces the need to treat ethnospecies as culturally relevant data, but taxonomically dependent on subsequent validation [2,35,36].

#### Botanical Resources, Plant Parts, and Ritual Functions

Plant uses were recoded from the consolidated plant spreadsheet (Supplementary Data S1, Tables S1–S13, and Supplementary Table S14). Because information on plant part, preparation, and ritual function was not described homogeneously across all studies, the analysis was organized into non-exclusive functional categories capable of capturing the overlap between pharmacobotanical materiality and ritual performativity.

The classification did not take the plant part alone as its main axis, because this field showed greater documentary distinction (Supplementary Table S14). Instead, each record was interpreted according to its function in the healing system, simultaneously considering therapeutic use, ritual support, form of preparation, spiritual mediation, protective purpose, diagnosis, and context of application. This methodological option avoids reducing the plant to an isolated drug and, at the same time, prevents ritual use from being marginalized or excluded from ethnopharmacological interpretation.

The recoded categories reveal strong overlap among performative, ritual, and pharmacobotanical dimensions. The most frequent category was “prayer, benzimento, and performative speech”, with 86 records, followed by “ingestion, tea, infusion, or decoction”, with 79, and “ritual, religious, or spiritual use”, with 75. Next were “pain, fever, inflammation, and infection”, with 67 records, “digestive, hepatic, and jaundice”, with 63, and “protection, cleansing, and descarrego”, with 41. This result supports the central argument of the article: plant knowledge in ritual healing contexts is not organized through an opposition between the pharmacological and the symbolic, but through the situated integration of these dimensions.

Brazilian records, especially those associated with benzimento in Chapada Diamantina and Santa Catarina, show that plants are combined with articulated sequences of words, gestures, intention, and ritual action. Species such as *Justicia gendarussa* Burm.f., *Alternanthera brasiliana* (L.) Kuntze, *Mangifera indica* L., *Coriandrum sativum* L., and *Dracaena trifasciata* (Prain) Mabb. [syn. *Sansevieria trifasciata* Prain] appear associated with prayers, baths, protection, energetic cleansing, inflammation, anxiety, agitation, and domestic uses [23,37]. These records allow benzimento to be interpreted as a botanical-performative practice, in which plant resources, ritual speech, and community care are articulated, and not merely as abstract prayer or domestic phytotherapy, as observed in Table 3.

Asian records show a strong presence of teas, infusions, decoctions, and oral preparations associated with jaundice, diarrhea, wounds, hypertension, fractures, diabetes, and digestive problems, often in contexts classified as traditional, spiritual, or medical-religious [38,39,40,41]. Within this set, the Solanaceae group requires particular analytical attention, because species of *Brugmansia* spp., *Nicotiana* spp., and *Datura* spp. may perform sacred, visionary, protective, or diagnostic functions while also presenting high chemical potency and biological risk. Their interpretation therefore requires an ethnopharmacological approach capable of simultaneously considering efficacy, symbolism, toxicity, and ritual context.

### 2.3. Descriptive Structure of Botanical Records and Specialist–Plant–Use Linkages

The botanical synthesis was organized as a traceable structure of source-coded links rather than as a comparative inventory of regional traditions. The consolidated matrix included 212 botanical entries, 68 botanical families, 166 genera, 44 source records or articles containing extractable botanical information, 24 canonical countries or regions, six continental groupings, 14 normalized specialist categories, and 13 non-exclusive use categories (Supplementary Table S14). Each value refers to documentary content in the assembled matrix; none represents the number of practitioners, communities, independent use events, or locally available species.

#### 2.3.1. Taxonomic Structure, Source Concentration, and Use Categories

The botanical matrix combines a concentrated taxonomic core with a long tail of less frequently recorded families (Figure S1; Table 2). Lamiaceae, Asteraceae, Solanaceae, and Fabaceae were the most represented families, but country-level totals were strongly affected by source-level extraction density. One Brazilian study contributed 40 of the 212 botanical entries (18.9%), and the five largest plant-list sources together contributed 99 entries (46.7%) (Supplementary Table S8; Figure S3). Brazil, India, and Ecuador therefore appear as documentary poles because the included sources from these settings supplied comparatively detailed plant lists, not because the review established greater local floristic richness or cultural importance (Figure S2).

Use categories also demonstrate overlap among ethnopharmacobotanical, ritual, and cosmological dimensions. General medicinal/therapeutic use crosses the entire normalized matrix, but appears associated with preparation or material support, rituality, spirituality, orality, benzimento, baths, teas, and protection. This convergence confirms that the plant, in these systems, rarely operates as an isolated object: its efficacy is combined and recognized through modes of preparation, gestures, words, regimes of authority, and culturally situated interpretations of illness [21,23,24,35,36].

#### 2.3.2. Record-Level Co-Documentation of Specialist Labels and Plant-Use Categories

This analysis addresses a restricted documentary question: within the assembled plant matrix, which original specialist denominations and non-exclusive plant-use categories were linked to the same source-coded botanical entries? The unit of analysis is a coded link connecting a botanical entry, its bibliographic source, the specialist denomination reported in that source, and one or more documented use categories. A cell or edge therefore indicates co-documentation in the matrix; it does not indicate that two practices co-occurred in a community, that a use was frequent among participants, or that specialist categories are culturally equivalent.

The magnitude of these links is conditioned by two measurable properties of the corpus: the number of records assigned to each label and the botanical detail of those records. The translated label “traditional healer” occurred in 46 of 186 analytical records (24.7%), whereas benzedor(a)/rezador(a) occurred in 10 (5.4%) (Supplementary Table S3). Nevertheless, benzedor(a)/rezador(a) accumulated 65 species–article attributions because several associated Brazilian studies contained extensive plant lists; “traditional healer” accumulated 74, “healer” 33, and yachak/yachakkuna 32 (Supplementary Table S7). Link magnitude is therefore a compound measure of label frequency and source-level botanical resolution, not a measure of cultural prominence.

Across the plant matrix, the most frequently coded non-exclusive domains were prayer, blessing, and performative word (86 entries); ingestion, tea, infusion, or decoction (79); ritual, religious, or spiritual use (75); pain, fever, inflammation, or infection (67); digestive, hepatic, or jaundice-related use (63); and protection or cleansing (41) (Supplementary Table S10). Their overlap shows that the reviewed sources often describe plants simultaneously as therapeutic preparations and as components of performative or ritual care. The specialist-by-use heat map has been transferred to Supplementary Figure S12, where it is retained as an audit of the coding structure rather than as a cross-cultural comparison.

### 2.4. Record-Level Documentation of Ritual-Architecture Domains

To remove the unsupported regional comparison, ritual architecture was reanalyzed at the level of the 186 analytical records. Each source was coded independently for seven non-exclusive domains: word or prayer; gesture over the body, object, plant, or space; material supports; mediation with entities; protection or cleansing; an explicit link to a broader religious or cosmological tradition; and symbolic or oracular diagnosis. High indicated central and explicit documentary emphasis; Medium indicated secondary or episodic presence; Low indicated absence, marginality, or little relevance in the source description; and Undetermined indicated insufficient information. The revised analysis reports the distribution of these codes across records and no longer collapses them into continental profiles.

**Figure 1:**
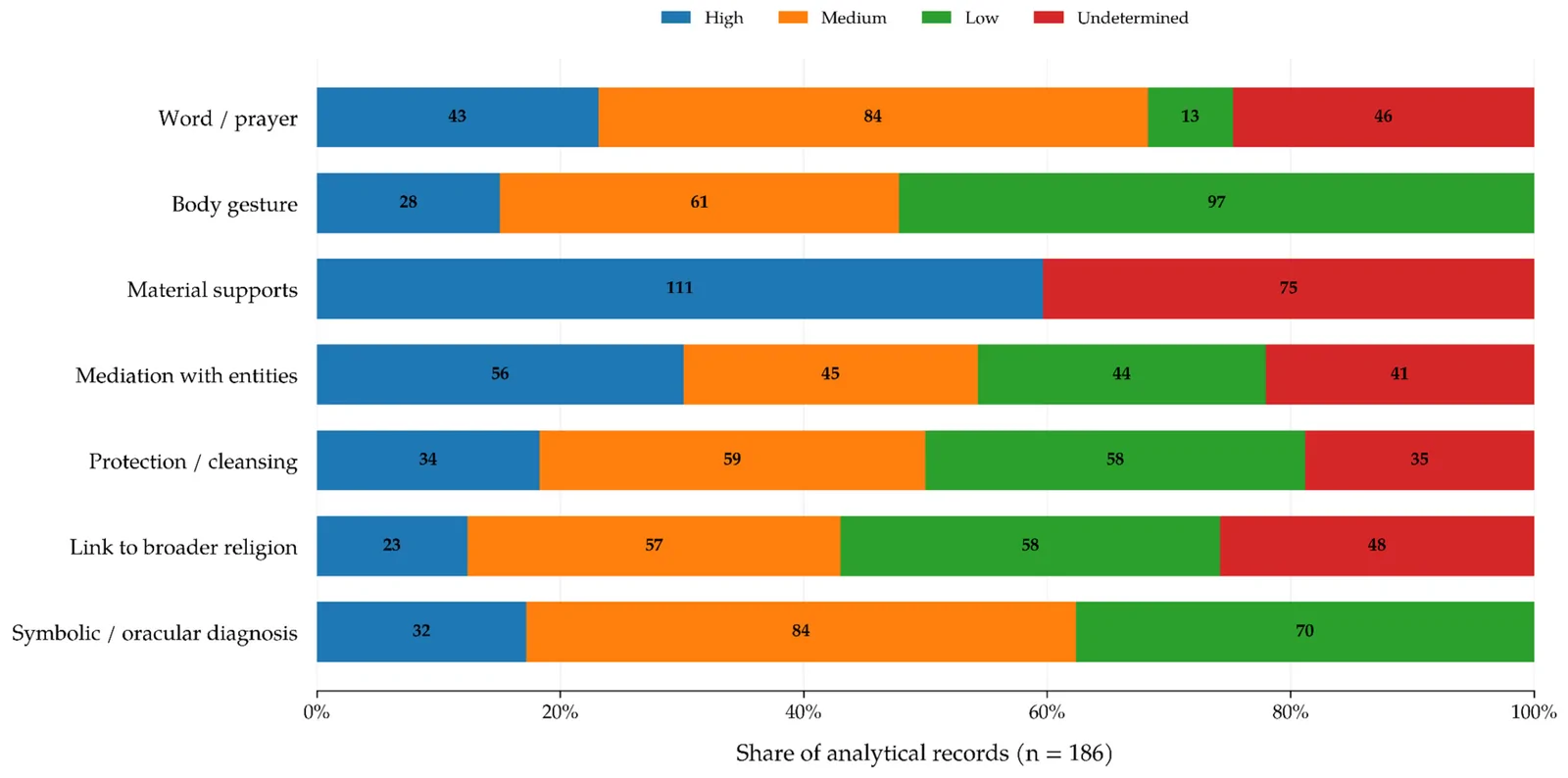
Record-level distribution of documentary-emphasis codes across seven ritual-architecture domains (*n* = 186 analytical records). Values inside segments are record counts. Percentages refer to reviewed sources, not practitioners, communities, regions, or cultural prevalence.

Word or prayer received High or Medium emphasis in 127 records (68.3%); symbolic or oracular diagnosis in 116 (62.4%); material supports were explicitly central in 111 (59.7%); mediation with entities received High or Medium emphasis in 101 (54.3%); protection or cleansing in 93 (50.0%); body gesture in 89 (47.8%); and explicit links to broader religious or cosmological traditions in 80 (43.0%) (Figure 1; Supplementary Table S4). Undetermined coding was especially frequent for material supports (75 records), broader religious links (48), word or prayer (46), and mediation with entities (41), identifying domains for which the reviewed sources often lacked sufficient description.

The record-level distribution supports a limited but defensible conclusion: verbal, material, spiritual, protective, gestural, and diagnostic dimensions are recurrently documented in the assembled literature, although the completeness and emphasis of reporting vary substantially among sources. These codes characterize the content of documents; they are not used to infer regional hierarchies, historical trajectories, cultural equivalence, or the frequency of practices in the underlying communities.

### 2.5. In Gesture, Word, and Faith: Semantic and Semiotic Reading of Ritual Healing Acts

The semantic and semiotic reading of benzimento requires shifting the analysis from the naming of specialists to the structure of the ritual healing act. Categories such as “benzedeira”, “machi”, “sangoma”, “hakim”, “ngangkari”, or “curandera” do not designate interchangeable positions, but rather forms of therapeutic authority situated in specific historical, territorial, and cosmological contexts (Table 4). Each term mobilizes its own conceptions of personhood, diagnosis, illness, protection, gift, secrecy, occupied territory, ancestry, and legitimacy, referring to a particular grammar of intervention upon suffering. Thus, cross-cultural comparison does not presume equivalence among specialists, but examines whether healing is produced through acts, by articulating word, gesture, matter, faith, spiritual agency, and community recognition. In these processes, ritual elements do not function as accessories, but as semiotic and performative operators that make the passage from vulnerability to protection, purification, repair, and therapeutic restoration intelligible, perceptible, and socially recognizable [8,12,42,43,44].

Translational terms such as “faith healing”, “prayer healing”, “spiritual healing”, “healing ritual”, and “shamanic healing” may be useful for locating comparative patterns, but they are insufficient as final interpretive categories when they do not preserve local names and the ritual sequences described in the studies. Part of the distinct emic precision that informs the social position, form of authority, transmission regime, therapeutic cosmology, and territorial bond of these agents is lost. The term shaman, in particular, requires caution, because its expansion as a global label for practices of trance, spiritual mediation, and diagnosis may produce improper equivalences among distinct historical and cosmological systems [45].

Thus, the cross-cultural comparison focused on documented acts--praying, blessing, cleansing, fumigating, blowing, touching, bathing, symbolically cutting, or mediating spiritual agencies--rather than presuming that generic categories describe equivalent practices [46,47,48,49,50,51]. Therefore, preserving native naming, accompanied by analytical contextualization, is essential to avoid undue homogenization, semantic displacement, and the false impression that heterogeneous ritual systems express the same universal category of healing [8,12,42].

In the analyzed corpus, the act of benzer was primarily understood as a ritual structured through the articulation of diagnosis and a healing procedure that combines gesture, word (prayer, reza, blessing, chant, verbal formula), and faith within the ritual practice, with or without the incorporation of auxiliary material elements, as described in Table 2. Branches, leaves, water, oils, ashes, smoke, foods, threads, scissors, or other objects may participate in the therapeutic action, but their presence alone does not define the act of benzer. The semantic core of the act lies in the performative capacity of ritually authorized speech, guided by intention, community recognition, and spiritual mediation, whether named as an entity or simply as faith. Thus, when present, plant or object materiality acts as a mediating support for healing, without replacing the centrality of enunciation, gesture, and faith in the ritual production that leads to healing.

**Table 4:** Comparative semantic and semiotic reading of selected healing terms.

Continent/Reference Region	Specialist	Associated Term/Practice	Main Semantic Focus	Semiotic Value in the Healing Act	Comparative Caution
South America, especially Brazil	Benzedeira/benzedor	Benzimento; benzer	Blessing, protection, ritually authorized speech, faith, and therapeutic gesture.	Performative act in which word, intention, and gesture symbolically reorganize vulnerability. Plants, water, oil, smoke, ash, or signs may integrate the ritual scene as semiotic supports of the action.	Do not reduce it to prayer, phytotherapy, or generic “spiritual healing”. Benzimento should be read as a situated practice, frequently linked to Brazilian popular Catholicism and to Indigenous and Afro-diasporic recompositions of care [8,12,52].
South America, especially Brazil	Rezadeira/rezador	Reza; healing prayer	Prayer, devotional recitation, intercession, and ritual speech.	Praying speech constitutes the most visible operator of efficacy, mediating request, protection, relief, and restoration.	Reza may be part of benzimento, but may also exist as an autonomous denominated practice; plants and objects may appear as support, not as a necessary axis.
South America, especially Brazil	Curandeiro/curandeira	Healing; curandeirismo; popular medicine	Broad therapeutic authority, plural care, and management of bodily, spiritual, or social suffering.	Healing may articulate counseling, plants, baths, prayers, bodily manipulation, and spiritual mediation, depending on the context.	Broad Portuguese category; it should be contextualized to avoid erasing differences among benzimento, raizeirismo, religious practices, domestic care, and popular therapeutics [12].
Latin America; North America in Mexican-American contexts	Curandero/curandera	Curanderismo; limpia; remedios; sobada; ritual diagnosis	Latin American popular healing, restoration of balance, diagnosis, and spiritual-material intervention.	The practice may combine plants, prayers, cleansings, massages, ritual objects, and relational explanations of illness.	Do not translate simply as “healer”. The term has its own historicity in Latin American and Mexican-American contexts, with strong regional variation.
South America, especially Brazil; Afro-Atlantic diaspora	Pai de santo/mãe de santo. Babalorixá/ialorixá.	Orixa priesthood; spiritual consultation; bath; ebó; ritual work; obligation	Afro-Brazilian religious authority, mediation with orixás, entities, or sacred forces, axé, and restoration of balance.	Healing articulates spiritual diagnosis, leaves, offerings, ritual prescriptions, community belonging, and restoration of relations among person, body, and the sacred.	This is a religious authority situated in Afro-Brazilian systems, with its own liturgy, hierarchy, and transmission regimes [53,54].
South America, especially Brazil	Raizeiro/raizeira	Use of roots, barks, leaves, garrafadas, and homemade remedies	Botanical-therapeutic knowledge, collection, preparation, and indication of medicinal plants.	The medicinal plant is the main sign of competence, articulating territory, empirical experience, local memory, and community circulation of knowledge.	Closer to ethnobotanical specialization, although there may be overlap among plants, prayers, and spiritual care.
Latin America; North America in Latino-diasporic contexts	Yerbero/yerbera; yerbatero/yerbatera	Use, sale, indication, and combination of medicinal herbs	Plant specialization, popular markets, circulation of remedies, and therapeutic counseling.	Authority is expressed in the selection, combination, and indication of herbs as material resources of care.	May designate a trader, plant specialist, therapeutic counselor, or ritual mediator, depending on the context [55].
Europe	Cunning man/cunning woman	Cunning craft; folk healing; counter-magic	European folk magic, domestic healing, protection, divination, and management of misfortunes.	Authority derives from community reputation, formulas, objects, secrets, and practical knowledge about harmful forces and protection.	Functionally comparable to blessing and charming traditions, but should not be taken as the single historical origin of benzimento [56].
Europe	Wise woman	Folk healing; domestic healing; herbal and ritual care	Female community knowledge, domestic care, plants, childbirth, protection, and counseling.	Healing is organized through the articulation of experience, gender, local reputation, and practical-ritual knowledge.	Should not be automatically merged with cunning woman. The expression may designate different regimes of female knowledge, from midwives to domestic healers.
Europe	Charmer	Charm healing; verbal charms	Verbal charm, ritual formula, secrecy, and healing by word.	The formulated word acts as ritual technology: it names, displaces, cuts, or neutralizes harm.	Approaches benzimento through the centrality of ritual speech, but should be maintained in its own linguistic and historical context [8].
Europe, especially Francophone contexts	Panseur/panseuse de secret	Pansement de secret; healing secret	Secret verbal formula, restricted transmission, and healing through interdiction or symbolic sealing.	Secrecy functions as a condition of authority and efficacy; healing operates through the combination of reserved speech, gesture, and ritual legitimacy.	The proximity to benzimento is semiotic, not genealogical. Do not translate simply as benzedor, prayer healer, or charmer.
Europe, especially Italy	Segnatore/segnatrice	Segnatura; segnare	Ritual signs, secret formulas, popular Catholicism, and gestural-verbal healing.	The sign, often associated with the cross or codified gestures, makes the intervention upon evil, pain, or vulnerability visible.	Do not translate automatically as benzedor. The comparison should preserve Italian regional specificity and the relationship among gesture, secrecy, and popular Catholicism.
Eastern Europe, especially Poland/Belarus	Szeptucha/szeptun	Szeptanie; whisper healing	Whisper, prayer, secrecy, intercession, and Slavic popular healing.	The low or whispered word acts as a form of mediation among suffering, faith, and symbolic restoration.	Translations such as “whisperer” or “folk healer” are insufficient; the term should be maintained with its religious, regional, and linguistic inscription.
Asia, with transregional diffusion as an analytical category	Shaman	Shamanic healing; trance; spiritual journey; diagnosis	Mediation with spirits, trance, ecstatic journey, diagnosis, and cosmological transformation.	The specialist operates as mediator between humans and non-human agencies, reorganizing relations among body, territory, spirit, and social order.	Term historically associated with Siberian/Tungusic contexts; its global use may erase emic categories and produce improper equivalences [45,57].
South America, especially Mapuche territory in Chile and Argentina	Machi	Machitún; ritual chant; diagnosis; Mapuche healing	Mapuche ritual authority, spiritual mediation, chant, plants, body, and territory.	Healing articulates body, spiritual forces, chant, plants, and sociocosmological recomposition.	Do not subsume generically under “shaman”. The category should preserve its Mapuche specificity, including gender, ritual power, and territoriality [58].
South America, especially Andes/Amazon	Yachak/yachakkuna	Limpia; diagnosis; ritual healing; knowledge	Situated knowledge, diagnosis, spiritual mediation, and Andean/Amazonian authority.	The therapeutic act involves knowledge, territory, plants, spirits, and community legitimacy.	Translations such as “shaman” or “traditional healer” reduce the emic density of the term; the local category should be preserved whenever possible.
Africa, especially Southern Africa	Sangoma	Ukubhula; ancestral diagnosis; ritual healing	Ancestry, spiritual calling, divination, diagnosis, and social recomposition.	Healing operates through mediation with ancestors, reading of signs, counseling, and, in certain contexts, use of medicinal substances.	Do not confuse with inyanga. In South African contexts, sangoma refers more to divination and ancestry, whereas inyanga is closer to plant/material medicinal knowledge [59].
Africa, especially Southern Africa	Inyanga	Muthi; plant medicine; therapeutic preparation	Medicinal knowledge of plants, roots, barks, minerals, and therapeutic substances.	Authority is expressed in the preparation and indication of traditional medicines, frequently after diagnosis performed by the practitioner or articulated with other specialists.	Should not be treated as synonymous with sangoma; the distinction between ancestral divination and plant medicinal competence is analytically relevant.
Africa, especially Central Africa	Nganga	Ritual healing; protection; power objects; spiritual mediation	Central African ritual authority, spiritual power, protection, healing, and community recomposition.	Healing involves ritualized objects, ancestors, spiritual forces, body, and social order.	Avoid colonial translations such as “witch doctor”. The term requires precise historical and linguistic contextualization [60,61].
West Africa; Afro-Atlantic diaspora	Babalawo	Ifá; oracular consultation; ritual prescription	Ifá priesthood, divination, destiny, guidance, and restoration of balance.	Healing emerges from oracular interpretation and ritual prescription, articulating destiny, conduct, offering, and relationship with òrìṣà.	This is a specific divinatory and religious authority [62].
West Africa, especially Igbo context	Dibia	Afa; divination; protection; ritual healing	Igbo ritual specialist, spiritual diagnosis, protection, and repair of disorders.	The therapeutic act may articulate divination, ritual objects, plants, and recomposition of personal or community imbalances.	Translations such as “native doctor” or “witch doctor” are reductive; the term should be situated within Igbo cosmology.
Asia, especially South Asia	Vaidya	Ayurveda; diagnosis and Ayurvedic treatment	Codified medical system, textual tradition, lineage, diagnosis, and plant/mineral therapeutics.	Authority derives from texts, training, clinical observation, doṣa theory, and pharmacopeia. It treats mind, body, and spirit.	Differentiate from local ritual specialists. Although rituality may be present, it is a learned and institutionalized medical system in many contexts [63,64].
Asia, especially South Asia and transregional Islamic tradition	Hakim	Unani; tibb; humoral diagnosis	Greco-Arab-Islamic medicine, humoral theory, diet, pharmacopeia, and literate authority.	Healing is structured through the reading of temperament, humoral balance, and prescription of substances, diet, and life regimens.	Do not group generically with ritual healers; its authority belongs to a textual and historical-institutional medical tradition [65].
Asia, especially South Asia	Kaviraj	South Asian popular/phytotherapeutic medicine	Plant knowledge, local formulations, and popular therapeutic care.	Authority materializes in mastery of plants, preparations, and therapeutic indications, often outside formal biomedicine.	Does not automatically correspond to vaidya; it may designate popular practitioners with different degrees of formalization and recognition.
Asia, especially Himalaya/Tibet	Amchi	Sowa-Rigpa; Tibetan medicine	Tibetan textual tradition, diagnosis, plants, minerals, diet, and medical-Buddhist cosmology.	Healing articulates body, environment, morality, medicinal substances, and literate tradition.	Differentiate from shamans and local ritual healers; this is a codified medical system, although cosmologically situated [66,67].
Asia, especially the Philippines	Albularyo/albulario	Orasyon; hilot; use of herbs; spiritual diagnosis	Filipino popular healing, prayer, herbs, touch, oils, and supernatural explanations of illness.	Authority combines plant knowledge, prayer, bodily manipulation, and spiritual diagnosis.	Does not simply correspond to herbalist or faith healer; it occupies a plural zone among herbs, prayer, touch, and popular spirituality.
Oceania, especially Central Australia	Ngangkari	Ngangkari healing; touch; symbolic extraction; spiritual care	Australian Aboriginal healing, body, spirit, territory, ancestry, and relational care.	Efficacy is anchored in the alignment among spirit, body, belonging, territory, and cultural protocols.	Do not reduce to “spiritual healer”. The term is linked to specific Indigenous systems of knowledge, care, and cultural governance.
Oceania, especially Aotearoa/New Zealand	Tohunga	Karakia; ritual knowledge; rongoā; specialized authority	Māori specialist, genealogy, mana, tapu, karakia, and situated knowledge.	Healing depends on ritual authority, sacred speech, ancestral relations, cultural protocols, and relational balance.	Do not translate generically as “priest” or “healer”. Tohunga may designate specialists in different domains of Māori knowledge [68,69].
Oceania, especially Aotearoa/New Zealand	Rongoā practitioner/rongoā healer	Rongoā Māori; karakia; use of plants; mirimiri	Māori healing, medicinal plants, spirituality, body, territory, and whakapapa.	The medicinal plant integrates a relational healing system, inseparable from karakia, protocols, ancestry, and territorial belonging.	Avoid extracting botanical lists from their spiritual, political, and relational contexts; rongoā is not merely phytotherapy [68,69,70].
Oceania, especially Hawaiʻi/Polynesia	Kahuna lāʻau lapaʻau	Lāʻau lapaʻau; pule; lāʻau; diagnosis and plant preparation	Traditional Hawaiian healing, plants, prayer/intention, genealogy, balance, and ancestral knowledge.	Efficacy articulates plant, word, spirituality, body, community, and collection/preparation protocols.	Do not reduce to herbalist. Lāʻau lapaʻau integrates plant knowledge and spirituality in a historical context marked by coloniality, regulation, and medical pluralism [71].

### 2.6. Transmission, Legitimacy, and Community Authority

The documentary corpus establishes that the transmission of ritual healing knowledge is not equivalent to the transmission of a list of plants. The literature indicated multiple pathways: family learning, observation, practical apprenticeship, religious initiation, dreams, experiences of illness, spiritual calling, secrecy, oral memory, and community participation [46,47,48,72,73,74,75]. In many traditions, knowing the name of the plant or prayer is insufficient. It is necessary to know when, how, for whom, and under what conditions care should be performed, which involves mastery of the ritual sequence, mode of preparation, gestures, restrictions, the moral and spiritual disposition of the recognized authority, and the concrete situation of suffering in which the intervention becomes appropriate [46,47,51].

Gender is also a central dimension in the transmission and legitimization of ritual healing systems. Many practices of prayer, blessing, use of medicinal plants, and domestic care are associated with women, especially older women, mothers, grandmothers, and family or community care networks [76,77,78]. This does not mean that ritual healing is exclusively female, since there are male specialists, priests, diviners, shamans, herbalists, and therapists linked to codified medical systems [79,80,81,82,83]. However, the recurrence of women as guardians of prayers, backyard plants, therapeutic memories, and everyday care shows that an important part of the community health infrastructure operates outside the formal institutions of medicine and the State [84,85].

Contemporary threats to the continuity of ritual healing knowledge include urbanization, migration, formal schooling, religious intolerance, biomedicalization, hybridization, environmental degradation, habitat loss, aging of practitioners, and changes in modes of intergenerational transmission, thereby modifying the recognition, use, and management of medicinal plants, displacing local repertoires and increasing dependence on introduced or commercialized species [48,75,86,87,88]. Similarly, processes of migration, socio-spatial transformation, and biodiversity loss challenge situated forms of learning based on coexistence, practice, territoriality, and transmission across generations [89]. These pressures, however, do not necessarily imply the disappearance of ritual healing; they may produce displacements, recompositions, and hybrid forms of continuity in domestic, community, religious spaces, or at interfaces with formal health systems. Thus, the analysis should avoid both the romanticization of tradition as an immutable inheritance and its disqualification as an archaic residue of the past. Rather, these practices should be understood as transforming practices crossed by vulnerabilities, disputes over legitimacy, and situated strategies of permanence [90].

### 2.7. Medical Pluralism, Public Health, and Epistemic Recognition

The findings situate ritual healing within the field of medical pluralism, understood as situated circulation among agents, spaces, therapeutic technologies, and explanatory regimes of illness. This circulation does not correspond merely to an abstract coexistence between biomedicine and traditional practices, but to everyday negotiation among health posts, hospitals, pharmacies, homes, plant markets, religious spaces, prayer networks, and ritual healing specialists. In the analyzed corpus, this pattern appears explicitly in studies on Latin American, African, and Asian communities, in which therapeutic choice depends on access, cost, trust, perceived severity, previous experience, attributed efficacy, and cultural interpretation of suffering [91,92,93,94,95]. Thus, the combined use of biomedicine, medicinal plants, prayer, counseling, symbolic diagnosis, protection, and community care should be interpreted as a pragmatic care itinerary, and not as a therapeutic contradiction.

This reading is particularly relevant because different studies in the corpus show that ritual healing specialists operate in interface zones among bodily care, social suffering, spiritual explanation, and community vulnerability. In Bangladesh, traditional practices were described as an integral part of local care systems and as responses to therapeutic demands not fully absorbed by formal medicine [92]. In Equatorial Guinea, Fang traditional medicine was associated with both medical and cultural afflictions, mobilizing specialists, community leaders, health professionals, and family members within an informal field of care seeking [95]. Among Garo women in India, the cultural persistence of traditional practices appears linked to situated decisions of therapeutic seeking, especially in contexts in which gender, access, and trust modulate the relationship with formal services [94]. These examples indicate that local categories of suffering cannot be reduced to residual beliefs: they organize the reading of illness, the choice of specialist, and the legitimacy of the intervention.

The relationship between ritual healing specialists and public health therefore remains ambivalent. On the one hand, these agents may function as community mediators of care, especially in territories where access to the formal system is limited, irregular, or culturally insufficient. Studies with Bapedi healers, traditional healthcare providers in Rajasthan, Zeliangrong communities of northeastern India, and rural South African groups show that these specialists may act in everyday primary care, knowledge transmission, management of medicinal plants, biocultural conservation, and responses to common health conditions [40,96,97,98]. On the other hand, the same interface may produce tensions, especially when traditional practices are evaluated solely by biomedical parameters, disregarding community authority, local cosmology, ritual protocols, quality of ethnobotanical documentation, and risks associated with inappropriate plant use or delayed biomedical care in severe cases.

#### Legal, Religious, and Institutional Vulnerability

Beyond clinical and epistemic tensions, ritual healing specialists may face systemic legal and institutional risks. Historical suppression, including the Tohunga Suppression Act in Aotearoa/New Zealand, illustrates how state regulation has criminalized or delegitimized Indigenous healing authorities [87]. In other contexts, practitioners may encounter religious discrimination, racialized stigma, accusations of witchcraft or charlatanism, exclusion from formal referral networks, restrictions on sacred practices, and appropriation of knowledge without recognition or benefit-sharing [90,99]. These pressures are not peripheral to therapeutic itineraries: they shape whether specialists can practice openly, transmit knowledge, collect or manage plants, collaborate with health services, and retain community authority. Medical pluralism should therefore be analyzed not only as pragmatic coexistence, but also as an unequal field structured by law, institutional power, racism, and religious freedom.

From an analytical perspective, epistemic recognition requires treating “ritual healing specialists” as a relational category, not as a universal synonym for “traditional healer”. The corpus reveals the coexistence of broad labels of academic translation, such as traditional healer and healer, with emic denominations of greater sociocultural density, such as benzedor(a)/rezador(a), yachak/yachakkuna, curandero/curandera, sangoma, vaidya, hakim, albularyo, dukun, tohunga, and rongoā healer. These categories condense different regimes of authority, transmission, diagnosis, spiritual mediation, relationship with plants, territory, and community recognition [24,25,26,75,100]. Therefore, cross-cultural comparison should operate through limited, domain-based comparison: the documented therapeutic function, ritual architecture, position of the plant, and community authority are compared without presupposing ontological equivalence among distinct historical, religious, and territorial traditions.

For ethnopharmacology and public health, this point shifts the unit of analysis from the isolated plant to the specialist-plant-ritual-territory-local illness category complex. The plant species should be interpreted together with vernacular name, taxonomic identification, plant part used, mode of preparation, route of administration, ritual context, native category of use, specialist authority, community circulation, and quality of documentary evidence. Studies with Saraguro yachak/yachakkuna, curandeiros/curanderas, mãe/pai de santo, Brazilian healers, vaidya, and Asian traditional specialists show that plants may operate simultaneously as pharmacological resources, ritual supports, territorial markers, spiritual mediators, instruments of protection, and performative elements of healing [24,25,26,41,93,99]. Thus, documented efficacy should not be mechanically separated from the word, gesture, faith, symbolic diagnosis, and social recognition that structure therapeutic practice.

The interface with public policies should therefore prioritize intercultural dialogue, safety, consent, community autonomy, and protection against misappropriation. This includes training health professionals to recognize plural therapeutic itineraries, qualified communication about the concomitant use of plants and medicines, identification of clinical warning signs, respect for ritual secrets and transmission restrictions, as well as ethical mechanisms for benefit-sharing when traditional knowledge informs research or products. The challenge is not to incorporate ritual healing uncritically into biomedical systems, nor to subordinate it to external criteria that erase its own logic, but to build zones of translation, safe care, and epistemic justice in which different rationalities can coexist without folklorization, criminalization, or instrumentalization.

### 2.8. Ritual Healing Specialists as Mediators of Plant-Based Community Knowledge

The consolidated results support an integrative model in which ritual healing specialists act as mediators of plant-based community knowledge, and not as isolated holders of botanical information (Table 5). Their practices connect leaves, branches, roots, water, smoke, oils, baths, foods, objects, words, hands, bodies, entities, ancestors, divinities, territory, memory, secrecy, and social legitimacy [23,46,47,51,75]. Plant knowledge therefore appears as part of a material, performative, relational, and political therapeutic ecology.

This model can be understood in four analytical layers (Table 5). The first is botanical material, formed by species, taxa, ethnospecies, plant parts, preparations, smoke, baths, oils, waters, foods, and ritual objects. The second is performative, composed of prayer, blessing, chant, whisper, sign, touch, blowing, hands, and ritual sequence. The third is relational-cosmological, involving spirits, ancestors, divinities, territory, moral order, and non-human agencies. The fourth is community-political, associated with legitimacy, gender, lineage, secrecy, memory, trust, interfaces with public health, cultural rights, and epistemic justice [8,10,21,35,36,100,101].

The central contribution of the review is twofold. First, it demonstrates that recurrent analytical domains occur across ritual healing practices in multiple cultural regions, while each system must be interpreted from its own categories, histories, and cosmologies; Brazilian benzimento is discussed as one situated configuration within this comparison. Second, it shows that the ethnopharmacology of plants in ritual contexts requires an expanded approach capable of integrating botanical identification, ritual sequence, specialist authority, local illness category, and community governance. Thus, leaves, prayers, gestures, healing, spirituality, and community are not separate dimensions; they form the same therapeutic architecture.

#### Implications and Priorities for Future Ethnobotanical Research on Ritual Healing Systems

The findings of this review indicate that ethnobotanical and ethnopharmacological studies on ritual healing specialists need to move beyond approaches restricted to medicinal plant inventories and generic translations of local categories. Lists of species, vernacular names, therapeutic indications, and preparation methods remain indispensable components of ethnobotanical documentation and characterization. However, ritual healing systems cannot be adequately understood when plants are analyzed separately from symbolism, prayers, gesture, diagnosis, spiritual mediation, protection, memory, territory, and community legitimacy. Within the reviewed literature, plants perform multiple documented roles within community systems of care, including pharmacological resources, ritual supports, diagnostic instruments, spiritual mediators, protective agents, and markers of biocultural belonging. Thus, future research should use integrated analytical matrices that relate taxon, vernacular name, plant part, preparation, mode of use, ritual sequence, specialist category, local theory of disease, form of knowledge transmission, and degree of community recognition [3,102,103,104].

The first weakness to be addressed is terminological imprecision. The broad use of expressions such as traditional healer, folk healer, spiritual healer, healer, or shaman may facilitate bibliographic search, but it also reduces complex local categories to universal equivalents. This practice erases important differences among therapeutic authorities, forms of initiation, social legitimacy, moral conduct, spiritual agency, ritual secrecy, lineage, belonging, and territorial particularities. Terms such as benzedeira/benzedor, rezadeira/rezador, curandero/curandera, sangoma, nganga, yachak, hakim, vaidya, albularyo, tohunga, kahuna, and ngangkari should be preserved as emic categories, and not treated merely as local variants of a universal figure of the “healer”. Future studies should retain original terms, present literal and contextual translations, describe the social position of specialists, and clearly differentiate emic categories, analytical categories, and terms used for bibliographic indexing [9,10,12].

The second weakness concerns low methodological standardization. Many studies still insufficiently describe the sampling strategy, participant profile, criteria for recognizing specialists, number of interviews, local validation procedures, linguistic mediation, ethical consent, botanical material collection, and taxonomic verification. This limitation compromises comparability among regions and reduces the reproducibility of ethnobotanical data. Future investigations should report whether specialists were identified through snowball sampling, community nomination, institutional recognition, ritual lineage, self-identification, or combined strategies. They should also clarify whether plants were identified using fertile botanical material, herbarium vouchers, photographic records, updated taxonomic databases, or only vernacular attribution. Whenever possible, voucher specimens, taxonomic authorities, herbarium numbers, and updated nomenclatural sources should be presented, especially when studies have pharmacognostic, conservation, or public health implications [105,106,107,108]. In light of this weakness, it is necessary to recall that, in certain contexts, the universe of locally recognized specialists may be small because of the social rarity of the function, restricted transmission of knowledge, aging of practitioners, low public visibility of the practice, processes of stigmatization, or the very community organization of these healing systems, which admits only a few specialists. In such cases, small samples may reflect the actual composition of the empirical field, and not necessarily methodological insufficiency. Thus, more important than requiring large numbers of participants is to make explicit the accessible universe, criteria for recognizing specialists, recruitment strategy, possible saturation, limits of generalization, and relationship among sample size, ethnographic depth, and study objective.

The third direction is to strengthen the articulation among ethnobotany, ritual studies, and medical anthropology. The reviewed literature shows that therapeutic efficacy in ritual healing systems is not only pharmacological. It is also relational, performative, symbolic, and socially recognized. Therefore, studies should not record only “which plant is used” and “for which disease”. They should document who uses the plant, under what ritual conditions, with which words, gestures, restrictions, prayers, offerings, diagnostic acts, spiritual agencies, and community expectations. This approach does not diminish the pharmacological interest of the species; on the contrary, it prevents complex therapeutic systems from being reduced to bioactivity screening and allows ethnopharmacology to incorporate symbolic, sensory, ecological, and social dimensions of healing [2,3,7,8].

The fourth weakness lies in the unequal distribution of evidence. The analyzed corpus reveals asymmetries of language, indexing, regional visibility, disciplinary coverage, and botanical resolution. Some traditions are described by detailed ethnographic and ethnobotanical studies, whereas others appear only through generic labels, secondary descriptions, or incomplete plant records. This review therefore does not claim to represent the totality of ritual-healing knowledge worldwide. Its cross-regional scope is limited to traceable records retrieved from Scopus, Web of Science, and the Virtual Health Library, together with a separately identified contextual layer; ethnographic, anthropological, regional, non-indexed, and literature in languages not covered by the search may remain underrepresented. South American records are especially prominent in the botanical matrix and were retained as a major interpretive axis, while records from other regions are used comparatively rather than as complete regional inventories. Future reviews should combine structured searches in international databases with manual tracking, reference chaining, and consultation of regional databases, keeping these sources methodologically differentiated. This distinction is necessary to avoid presenting heterogeneous evidence as if it resulted from a single standardized search strategy. It is also necessary to expand multilingual searches and include regional journals, especially from Latin America, Africa, Asia, and Oceania, where a relevant portion of ritual healing knowledge remains outside higher-impact international databases [107,108]. This expansion is particularly relevant because translation, indexing, and terminological harmonization may reduce the emic density of local names for specialists and their practices, causing historically situated categories to be described as equivalent regardless of their regional, cosmological, and therapeutic differences.

The fifth direction is ethical and political. Research with ritual healing specialists should be conducted with community participation, cultural safety, and a commitment to benefit-sharing. Many of these practices involve restricted knowledge, sacred plants, initiation rules, oral transmission, gender relations, community memory, and forms of secrecy that cannot be treated as freely extractable data. Future studies should adopt prior informed consent, collective validation, principles of data sovereignty, and clear criteria regarding what may or may not be published. This care is indispensable in contexts marked by religious intolerance, racism, colonial extraction, territorial loss, environmental degradation, and biomedical delegitimation of traditional knowledge. In this sense, ethnobotany should contribute not only to scientific documentation, but also to the protection of biocultural heritage, intergenerational transmission, territorial rights, and the epistemic recognition of these practices [10,78,90].

At this final stage, with faith and a gaze directed toward the future, subsequent studies should avoid transforming Brazilian benzimento into a universal model or default classificatory metric for ritual healing. Its value lies in serving as a situated Brazilian case through which relationships among word, gesture, plant, faith, protection, diagnosis, and community legitimacy can be discussed after independent domain-based analysis. Each healing system should be interpreted from its own cosmology, history, ecology, territory, and social organization. The most consistent direction for the field is to develop a relational and comparative ethnobotany of ritual healing, capable of preserving emic categories while identifying transcultural patterns in the articulation among plants, ritual performance, therapeutic authority, and community care. This agenda strengthens ethnobotany, ethnopharmacology, and public health by recognizing ritual healing specialists not as peripheral informants, but as central mediators of community knowledge about plants, biocultural memory, and plural therapeutic systems.

## 3. Materials and Methods

### 3.1. Study Design and Analytical Rationale

This study was designed as a cross-regional scoping review, with narrative synthesis and a descriptive-exploratory component. This methodological design was adopted because the object investigated is broad, interdisciplinary, terminologically heterogeneous, and distributed across different fields, including ethnobotany, ethnopharmacology, health anthropology, religious studies, cultural history, biocultural heritage, and public health. The scoping review was considered more appropriate than a systematic review of effectiveness or a meta-analysis, since the aim was not to estimate therapeutic effects, compare clinical interventions, hierarchize biomedical evidence, recommend the use of plant species, or claim exhaustive worldwide coverage [109,110,111].

The methodological conduct was guided by the Joanna Briggs Institute recommendations for scoping reviews and by the PRISMA-ScR extension, especially regarding explicit reporting of the research question, eligibility criteria, information sources, screening, extraction, categorization, and synthesis [107,112]. Considering the intercultural and terminologically fragmented nature of the topic, the review was organized into two methodologically distinct and analytically complementary layers: a formal scoping-review layer, derived from structured searches and subjected to the reported screening and operational eligibility pathway; and a contextual supplementary layer, composed of records incorporated through reference tracking and targeted searching by relevant emic categories [108].

This strategy was necessary because many ritual healing specialists are not adequately indexed in international databases or appear under generic translations, such as “traditional healer”, “folk healer”, “spiritual healer”, or “shaman”.

The two layers were not treated as methodologically equivalent. The formal layer followed the structured search, deduplication, title-and-abstract screening, and operational eligibility pathway reported in the PRISMA-ScR flow. The contextual supplementary layer did not originate from a single standardized search and was not subjected to an independent blinded screening procedure identical to the formal layer. It was therefore excluded from PRISMA retrieval rates and from claims of search completeness, regional prevalence, or exhaustive coverage, while remaining available for transparent contextual and terminological comparison.

### 3.2. Research Question, Objectives and PCC Framework

The guiding question was structured according to the PCC framework [112]. The population comprised rural, urban, Indigenous, Afro-descendant, quilombola, peasant, peri-urban, traditional communities, and other groups in which community healing practices are socially recognized. The concept included ritual healing specialists and plant-based community knowledge associated with Brazilian benzimento and other culturally situated practices, such as benzedeiras, benzedores, rezadeiras, rezadores, curanderos, curanderas, sangomas, ngangas, yachak/yachakkuna, machi, cunning folk, kahuna, ngangkari, tohunga, bomoh, dukun, babalawo, raizeiros, pajés, albularyos, and other local categories. The context encompassed popular, religious, spiritual, ritual, traditional, and community healing practices described in different countries and regions.

The main question was: how does the traceable scientific literature describe culturally situated ritual healing specialists in different regional contexts, how are plants mobilized in their care practices, and which analytical domains recur across these systems? Based on this question, five operational objectives were defined: to map the terminological diversity of specialists; to code plant use, speech, gesture, material support, protection, spiritual mediation, diagnosis, and community authority independently of any single tradition; to identify recurrent ritual structures; to consolidate botanical repertoires and plant-use categories; and to construct an integrative synthesis capable of relating plants, words, gestures, spirituality, territory, community legitimacy, medical pluralism, and Brazilian benzimento as one situated comparative case.

### 3.3. Information Sources, Search Strategy and Corpus Construction

Structured searches were conducted in bibliographic databases effectively documented in the review process, including Scopus, Web of Science, and the Virtual Health Library. Search strategies were adapted to the syntax of each database, combining broad descriptors and culturally situated terms in English, Portuguese, Spanish, and other languages when available. Terms such as “traditional healer”, “folk healer”, “spiritual healer”, “faith healer”, “prayer healer”, “ritual healing”, “medicinal plants”, “ethnobotany”, and “ethnopharmacology” were used, combined with emic or regional categories such as “benzedeira”, “benzedor”, “rezadeira”, “rezador”, “curandero”, “curandera”, “sangoma”, “nganga”, “kahuna”, “ngangkari”, “tohunga”, “bomoh”, “dukun”, “albularyo”, “cunning folk”, “wise woman”, “charmer”, “panseur de secret”, “segnatore”, “ensalmador”, “saludador”, and “szeptucha”.

The structured bibliographic search identified 255 records. After removal of 8 duplicates, 247 unique records were assessed by title and abstract. Of these, 61 records were forwarded for operational eligibility assessment, of which 54 were retained as the formal corpus of the scoping review. Subsequently, 132 records were identified through reference tracking and targeted searching by emic terms and were maintained as a distinct contextual supplementary layer. The complete analytical dataset therefore comprised 186 records, but the formal PRISMA-derived scoping-review corpus remained 54 records (Supplementary Material Tables S1 and S2).

The contextual supplementary layer was incorporated because many culturally situated categories have low bibliographic retrievability, appear in regional studies that are not homogeneously indexed, or are replaced by generic labels in international databases. To ensure transparency and traceability, formal and supplementary records were maintained in separate source-provenance fields in the analytical matrix. For supplementary records, the matrix retained the available title or reference, authorship and year when reported, DOI or public source when available, specialist term, geographic context, reason for analytical inclusion, and linked categories. The complete structured search strategy by database, including search date, fields consulted, filters applied, Boolean combinations, and number of records retrieved, was made available in Supplementary Table S2; the botanical database is presented separately in Supplementary Table S14.

Databases initially considered but not effectively incorporated in a traceable way into the main review flow were not treated as executed sources in the formal corpus. This methodological decision was adopted to avoid overestimating the scope of the search and to preserve the reproducibility of the process.

### 3.4. Eligibility Criteria and Analytical Boundaries

Studies were included if they described ritual, spiritual, religious, popular, or traditional healing specialists and presented, explicitly or interpretably, at least one of the following dimensions: relationship with plants or plant resources; community therapeutic practices; ritual procedures; prayer, blessing, charm, gesture, or cleansing; spiritual, symbolic, or oracular diagnosis; knowledge transmission; community legitimacy or authority; or interface with biomedical systems and public health. Empirical studies in ethnobotany, ethnopharmacology, anthropology, collective health, and related areas were prioritized. Reviews, conceptual essays, and historical-ethnographic studies were retained when they contributed to the interpretation of categories, cultural trajectories, medical pluralism, or ritual efficacy.

For inclusion in the botanical matrix, an additional criterion was applied: the source had to document a botanical entity, identified at the species, genus, family, vernacular-name, or ethnospecies level, in direct association with a healing specialist or culturally situated healing practice. Specific uses, plant parts, preparations, and routes of application were recorded only when explicitly reported in the source. Animal, mineral, synthetic, and other non-botanical materials were excluded from the plant matrix, although the corresponding source could remain in the broader specialist corpus when relevant to the characterization of the healing system.

Records centered exclusively on experimental or clinical pharmacology without a relationship to community specialists were excluded; doctoral and master’s degree completion documents that were not indexed articles; lists of plants disconnected from traditional, spiritual, or ritual practices; studies in which the specialist category could not be identified; documents lacking minimal contextual information on country, group, practice, or use; duplicate texts; and sources that merely reproduced data already represented in more complete records [108].

### 3.5. Screening, Operational Eligibility Assessment and Management of Borderline Records

The formal-search references were imported into Rayyan [113]. Records were standardized according to title, authors, year, source database, DOI, journal, and available abstract. Screening of the formal layer was performed by title and abstract, with records classified as “include”, “exclude”, or “maybe”. Conflicts were resolved by consensus among reviewers. When necessary, the final decision was made after reassessment of the record and analysis of the fields available in the spreadsheet.

The eligibility stage, in this version of the database, had an operational character because it was based on available metadata and abstracts, without systematic full-text reading of all articles. Exclusions were recorded according to the main reason, including absence of relevant empirical or analytical data, absence of community context, absence of a clearly identified ritual specialist, strict biomedical focus, or peripheral mention of the term of interest. The identification, screening, eligibility, and inclusion flow was documented according to the PRISMA-ScR model, containing the number of records identified by database, duplicates, exclusions by title and abstract, records assessed for eligibility, and studies included in the final synthesis [107,108].

#### Separate Treatment of Contextual Supplementary Records

Supplementary records were retained only when a traceable title or reference and sufficient information on the specialist, geographical or cultural context, and analytical relevance were available. They were coded with a distinct source status and were used to expand emic terminology, contextual interpretation, and cross-regional comparison. They were not used to reconstruct the denominator of the formal search, calculate screening performance, or imply that the same double-independent screening process had been applied to both evidence layers. Botanical information from supplementary records was extracted only when a plant entity and its association with a specialist or culturally situated healing practice were explicitly documented.

### 3.6. Data Extraction and Structure of the Evidence Matrix

Data were extracted into a structured matrix designed to allow traceability among bibliographic source, cultural category, ritual practice, botanical record, and analytical interpretation. The organization of the matrix followed methodological recommendations for scoping reviews, in which extraction should be sufficiently flexible to accommodate heterogeneous evidence, but standardized enough to allow comparative synthesis, transparency, and reproducibility [107,109,110,112].

The matrix was divided into six analytical blocks. The first block gathered bibliographic data: author, year, title, type of study, journal, DOI when available, database or source of location, and record code. The second block gathered geocultural data: country, region, continent, community or group referred to, territorial scale, and contextual observations. This separation made it possible to preserve the situated dimension of the records, a central aspect in ethnobotanical, ethnopharmacological, and anthropological studies, in which knowledge about plants, healing, and therapeutic authority depends on ecological, historical, territorial, and sociocultural context [114,115,116,117].

The third block recorded information about specialists: original term, translation when present, harmonized category, therapeutic role, type of authority, and described relationship with plants, spirituality, or community care. This strategy was adopted to avoid premature replacement of emic categories by generic analytical labels, a recurrent limitation in cross-cultural comparisons of local medical systems and traditional healing practices [12,118].

The fourth block described ritual architecture: presence of prayer, blessing, performative speech, gesture, touch, blowing, laying on of hands, use of material supports, mediation with entities, cleansing, protection, reversal of harms, and symbolic, divinatory, or oracular diagnosis. The inclusion of these dimensions was guided by classical and contemporary approaches to ritual performativity, symbolic efficacy, embodiment, spiritual mediation, and the social production of healing [7].

The fifth block gathered botanical information: scientific name, vernacular name, ethnospecies, family, plant part, form of preparation, mode of administration, ritual function, therapeutic indication, and degree of taxonomic certainty. This structure was adopted because the comparability of ethnobotanical studies depends on adequate documentation of scientific names, local names, parts used, forms of use, cultural context, and level of taxonomic resolution [119,120].

For the revised botanical database, the fifth block was organized to preserve the name used in the consolidated matrix, the name cited in the source spreadsheet, botanical family, vernacular name or ethnospecies when extractable, taxonomic resolution, specific documented use or application, documentary qualification, specialist category, country or region, continent, bibliographic record code, and a cautious verification note. When plant part, preparation method, route of application, voucher, or accepted-name confirmation was not explicitly available in the source material used for extraction, the field was retained as not reported or requiring confirmation rather than inferred.

The sixth block recorded transmission, legitimacy, gender, community recognition, relationship with biomedicine, implications for public health, and notes of uncertainty or limitation. These dimensions were included because ritual healing specialists do not act only as informants about plants, but as mediators of therapeutic systems in which knowledge, authority, trust, spirituality, social memory, and medical pluralism are inseparable components [118].

### 3.7. Terminological Harmonization and Classification of Specialists

Terminological harmonization was carried out in two stages. In the first, the original designation used in each study was preserved, such as benzedeira, rezador, curandero, yachak, machi, sangoma, nganga, vaidya, hakim, ngangkari, albularyo, or cunning folk. This preservation was necessary to maintain emic specificity and avoid replacing local categories with generic labels, since native terms often condense particular conceptions of body, person, illness, authority, spirituality, territory, and therapeutic legitimacy [118].

In the second stage, a harmonized category was assigned to allow cross-cultural comparison among records using different languages, disciplinary traditions, and indexing systems. This harmonization was conducted as an analytical procedure, and not as an ontological equivalence among specialists. Cross-cultural comparison was therefore guided by the described therapeutic function and the documented ritual context, avoiding the treatment of categories such as “traditional healer”, “folk healer”, “spiritual healer”, or “shaman” as universal cultural units [45,118].

The harmonized category was defined based on five criteria: semantic proximity of the term; described therapeutic function; type of authority attributed to the specialist; relationship with plants, preparations, or plant resources; and presence of spiritual, ritual, diagnostic, or community mediation. When a broad term such as “traditional healer”, “healer”, “spiritual healer”, or “shaman” did not contain sufficient information for greater precision, it was retained as a peripheral or low-resolution category [10].

### 3.8. Independent Analytical Domains and Comparative Positioning of Brazilian Benzimento

The analytical domains were defined independently of Brazilian benzimento: plant use, performative speech, bodily gesture, material support, protection or cleansing, spiritual mediation, symbolic or oracular diagnosis, community recognition, and therapeutic or restorative purpose. Each source was read within these domains before the Brazilian case was discussed. Benzimento was subsequently presented as one historically situated configuration characterized in specific Brazilian sources by recurrent articulations of prayer or blessing, gesture, plant or material support, protection, faith, and community legitimacy [7,12,118].

The analysis considered the described practice rather than only the specialist label. Similar labels may designate distinct practices, whereas different names may describe partially overlapping acts such as protection, cleansing, blessing, spiritual diagnosis, mediation with entities, or restoration of relational balance. Such overlap was used to organize thematic discussion, not to establish cultural equivalence or genealogical proximity [12,118].

No record was assigned a degree of proximity to benzimento, and no tradition was classified as an expanded or reduced variant of the Brazilian case. Benzimento was discussed only after the source-level synthesis and was not used to score, rank, or position other practices. Sources with insufficient detail remained in the terminological or contextual synthesis without inferential recoding [45,51].

### 3.9. Coding of Ritual Architecture

Ritual architecture was coded according to seven non-exclusive dimensions: word, prayer, or performative enunciation; gesture performed over body, object, plant, or space; consecrated material support, including leaves, branches, roots, water, smoke, oils, salt, baths, foods, or objects; mediation with saints, spirits, ancestors, divinities, entities, or non-human agencies; function of protection, cleansing, descarrego, removal, or reversal of harms; explicit link with a broader religious or cosmological tradition; and symbolic, divinatory, visionary, oracular, or spiritual diagnosis [8,43,44,52,121].

Each dimension received a documentary-emphasis code at the level of the individual analytical record: High, when the domain was central and explicitly described; Medium, when it was present in a secondary, implicit, or episodic way; Low, when it was absent, marginal, or of little relevance to the described practice; and Undetermined, when the source did not provide sufficient information. The primary synthesis reports record-level counts and percentages for these four codes. Regional aggregation was removed because it compressed heterogeneous documents into apparent continental profiles without comparable sampling, population, or documentation denominators [107,112].

### 3.10. Botanical Data Processing and Plant-Use Categories

Botanical records were treated as documentary units of occurrence, and not as estimates of community prevalence, actual frequency of use, or ecological availability. This decision was adopted because ethnobotanical and ethnopharmacological studies differ in sampling design, ethnographic depth, collection effort, taxonomic identification, inclusion criteria, and the way plant uses are recorded [116,119]. In this review, a botanical entry was defined as a documentary record retained in the consolidated plant matrix. Entries could correspond to taxa identified at the species, genus, or family level, as well as vernacularly identified ethnospecies when no more precise taxonomic determination was available.

When scientific names were provided, they were extracted as presented by the original authors. Links, taxonomic databases, and resources such as Plants of the World Online, World Checklist of Vascular Plants, and World Flora Online were used as documentary support for checking and organizing names, without constituting automated taxonomic validation or full nomenclatural review of all records. Vernacular names and ethnospecies were preserved when botanical identification was incomplete [120,122]. Uncertain records were retained, but coded as taxonomically incomplete, ambiguous, or dependent on later validation [114,116,119,123].

The position of plants in ritual systems was classified into non-exclusive functional categories: central therapeutic axis, ritual support, spiritual or cosmological mediator, diagnostic or divinatory element, protective or cleansing agent, and peripheral or uncertain record. This classification was applied because the same plant may simultaneously operate as a remedy, ritual object, sensory mediator, cleansing instrument, symbolic marker, or component of a therapeutic sequence. This overlap among plant materiality, symbolism, spirituality, and community care is recurrent in ethnobotanical, ethnopharmacological, and anthropological studies on traditional medical systems [117,124].

Plant uses were recoded into non-exclusive categories derived from source descriptions: prayer, benzimento, and performative speech; ingestion, tea, infusion, or decoction; ritual, religious, or spiritual use; pain, fever, inflammation, or infection; digestive, hepatic, or jaundice-associated use; protection, cleansing, or descarrego; bones, fractures, or sprains; topical, bodily, or wound application; childbirth, puerperium, reproduction, or gynecology; baths, washes, or hydrotherapeutic uses; diagnosis, divination, vision, or oracle; mental suffering, fear, fright, or nerves; and smoke, fumigation, tobacco, or inhalation. The consolidated plant-level table, containing the source-cited and consolidated names, botanical family, vernacular or ethnospecies information when available, taxonomic resolution, associated specialists, countries or regions, ritual or therapeutic uses, documentary qualifications, and bibliographic record codes, was made available as Supplementary Table S14.

### 3.11. Descriptive Analyses and Exploratory Visual Synthesis

Descriptive analyses were performed from the curated matrices of specialists, ritual dimensions, and botanical records. Absolute and relative frequencies were calculated for continents, countries or regions, specialist categories, domain-overlap descriptors, botanical families, and plant-use categories. Because many studies contained multiple specialists, plants, uses, plant parts, or geographical attributions, the categories were treated as non-exclusive. Therefore, the totals presented in the results represent documentary attributions, not individuals, communities, independent therapeutic events, or population estimates. This distinction is fundamental in scoping reviews, especially when the data are heterogeneous, multireferential, and derived from different methodological designs [107,112].

Relational visualizations were retained only as supplementary data-audit tools, and their analytical units are now stated explicitly. Bar lengths count source-coded plant links; alluvial flow widths count matrix rows connecting categories; overlap sectors count unique taxon labels assigned to more than one continental grouping; bipartite edge weights count co-attributions; and treemap areas count nested documentary records. The main-text interpretation relies on record-level frequencies, article-level source concentration, and explicitly defined co-documentation counts rather than on visual comparison of regions [125,126,127,128].

No country- or continent-level flora total was used as a denominator because the primary studies sampled local, culturally selected plant subsets under incompatible designs and levels of taxonomic resolution. Instead of attempting post hoc ecological normalization, the revised analysis examines the observed source structure directly by reporting the contribution of individual articles to the plant matrix and by separating specialist records from botanical entries. Supplementary cross-regional displays are retained for traceability only and do not support rankings of regions, floras, or healing systems [107,111,112,116,119].

Descriptive analyses were conducted in a Python 3.9 environment, with support from NumPy (v1.23.5), pandas (v1.5.3), Matplotlib (v3.6.2), Seaborn (v0.12.2), SciPy (v1.10.0), scikit-learn (v1.2.0), NetworkX (v2.8.8), and Plotly (v5.11.0) [129,130,131]. These libraries were used for matrix curation, absolute and relative frequencies, record-level distributions, source-contribution summaries, co-documentation counts, and supplementary graphical audit outputs [129,130,131].

Part of the analytical organization, refinement of Python scripts, and standardization of graphical routines was supported by the artificial intelligence tool ChatGPT (GPT-5; OpenAI, San Francisco, CA, USA). The tool was restricted to technical-writing and computational support and did not replace data curation, matrix verification, scientific interpretation, or authorial responsibility. All scripts, figures, and interpretations were critically reviewed by the authors. Complementary audit visualizations are provided in Supplementary Section S3, Figures S5–S12, and Tables S11–S13 [129,130,131].

## 4. Conclusions

The consolidated results support an integrative model in which ritual healing specialists are mediators of plant-based community knowledge and not isolated holders of botanical information. Their practices connect species, ethnospecies, plant parts, water, smoke, oils, baths, foods, objects, words, hands, bodies, spirits, ancestors, divinities, territory, memory, secrecy, and social legitimacy. Plant knowledge therefore appears as part of a material, performative, relational, and political therapeutic ecology.

This model has four analytical layers. The first is botanical material: species, taxa, ethnospecies, preparations, smoke, baths, oils, waters, foods, and ritual objects. The second is performative: prayer, blessing, chant, whisper, sign, touch, blowing, hands, and ritual sequence. The third is relational-cosmological: spirits, ancestors, divinities, territory, moral order, and non-human agencies. The fourth is community-political: legitimacy, gender, lineage, secrecy, memory, trust, interface with public health, cultural rights, and epistemic justice.

The central contribution of the reformulated section is twofold. First, it identifies recurrent analytical domains across ritual healing practices in multiple cultural regions while preserving their own categories, histories, and cosmologies; Brazilian benzimento is treated as one situated comparative configuration rather than as a default metric. Second, it shows that the ethnopharmacology of plants in ritual contexts requires an expanded approach capable of integrating botanical identification, ritual sequence, specialist authority, local illness category, and community governance. The formal 54-record scoping-review corpus and the 132-record contextual supplementary layer must remain analytically differentiated, and the cross-regional findings should not be interpreted as exhaustive global coverage. Thus, leaves, prayers, gestures, healing, spirituality, and community are not separate dimensions; they constitute the same therapeutic architecture.

## Figures and Tables

**Table 1:** Integrated categories of the position of plants in ritual healing systems.

Position of the Plant	Operational Definition	Representative Specialists	Analytical Implication
Plants as a central therapeutic axis	Botanical knowledge, preparation, indication, dosage, and administration sustain the specialist’s authority.	Raizeiros; yerberos; yachak/yachakkuna; hakims; vaidyas; rongoā healers; traditional healers with detailed botanical lists.	High ethnopharmacological relevance when taxonomic validation and context of use are available.
Plants as ritual support	Leaves, branches, baths, waters, oils, smoke, or plant objects are activated by word, gesture, and recognition.	Benzedeiras; rezadores; curandeiros; practitioners of benzimento and blessing.	Avoids reducing the plant to an isolated drug and also avoids excluding it because it is associated with prayer.
Plants as spiritual mediators	Species connect diagnosis, vision, protection, cleansing, and relationships with entities or territories.	Yachak/yachakkuna; vegetalistas; ayahuasqueros; machi; nganga; sangoma; tohunga; ngangkari.	Requires relational interpretation, attention to toxicity, secrecy, and cultural governance.

**Table 2:** Main botanical families of the consolidated plant repertoire associated with ritual healing specialists, with recorded species/ethnospecies and predominant continental attributions.

Botanical Family	Plant Records	Percentage	Recorded Species or Ethnospecies	Predominant Continental Attributions	Representative Documented Uses
Lamiaceae	20	9.4%	*Clerodendrum glandulosum* Lindl.; *Dracocephalum polychaetum* Bornm.; Lamiaceae spp.; *Leonotis nepetifolia* (L.) R.Br.; *Mentha pulegium* L.; *Mentha spicata* L.; *Ocimum americanum* L.; *Ocimum basilicum* L.; *Ocimum gratissimum* L.; *Ocimum tenuiflorum* L.; *Rydingia persica* (Burm.f.) Scheen & V.A.Albert; *Salvia fruticosa* Mill.; *Teucrium polium* L.; *Vitex agnus-castus* L.; *Clinopodium* sp.; *Melissa officinalis* L.; *Coleus amboinicus* Lour.; *Coleus barbatus* (Andrews) Benth. ex G.Don; *Salvia rosmarinus* Spenn.; *Thymus serpyllum* L.	South America; Asia	Teas, infusions, and decoctions; digestive complaints; pain and inflammation; topical applications; aromatic baths; prayer, protection, and ritual cleansing.
Asteraceae	19	9.0%	*Ageratum conyzoides* L.; *Cota palaestina* Kotschy.; *Calendula officinalis* L.; *Eclipta prostrata* (L.) L.; *Gynura cusimbua* (D.Don) S.Moore; *Launaea acanthodes* (Boiss.) Kuntze; *Sphaeranthus senegalensis* DC.; *Gymnanthemum coloratum* (Willd.) H.Rob. & B.Kahn; *Gymnanthemum amygdalinum* (Delile) Sch.Bip.; *Achyrocline alata* (Kunth) DC.; *Ambrosia artemisioides* Meyen & Walp.; *Artemisia sodiroi* Hieron.; *Baccharis obtusifolia* Kunth; *Baccharis* sp.; *Baccharis trimera* (Less.) DC.; *Bidens pilosa* L.; *Andicolea thuyoides* (Lam.) Mayta & Molinari; *Solidago chilensis* Meyen; *Tagetes terniflora* Kunth	South America; Asia	Teas and infusions; digestive and hepatic complaints; fever, pain, and inflammation; topical and wound care; baths, cleansing, and ritual uses.
Solanaceae	16	7.5%	*Brugmansia aurea* Lagerh.; *Brugmansia × candida* Pers.; *Brugmansia sanguinea* (Ruiz & Pav.) D.Don; *Brugmansia* spp.; *Brugmansia suaveolens* (Humb. & Bonpl. ex Willd.) Bercht. & J.Presl; *Capsicum* sp.; *Cestrum laevigatum* Schltdl.; *Nicotiana rustica* L.; *Nicotiana* spp.; *Nicotiana tabacum* L.; *Physalis halicacabum* Crantz; *Solanum incanum* L.; *Solanum lycopersicum* L.; *Solanum nigrum* L.; *Solanum oblongifolium* Dunal; *Solanum tuberosum* L.	South America; Asia	Medicinal and food uses; sacred and visionary applications; diagnosis or divination; smoke, fumigation, and tobacco-based practices; protection and ritual cleansing.
Fabaceae	12	5.7%	*Afzelia quanzensis* Welw.; *Cajanus cajan* (L.) Huth; *Hymenaea stigonocarpa* Mart. ex Hayne; *Indigofera antunesiana* Harms; *Piliostigma thonningii* (Schumach.) Milne-Redh.; *Poiretia bahiana* Cl.Müll.; *Prosopis farcta* (Banks & Sol.) J.F.Macbr.; *Senna occidentalis* (L.) Link; *Senna* sp.; *Bauhinia forficata* Link; *Pterodon emarginatus* Vogel; *Stryphnodendron adstringens* (Mart.) Coville	South America; Africa	Pain and inflammatory conditions; wounds and topical care; digestive complaints; medicinal and food preparations; protection and ritual applications.
Unidentified	9	4.2%	alecrim-de-vaqueiro; ares-da-felicidade; marinheiro; relógio (Andaraí); relógio (Lençóis); salva-de-ogum; tira morfina; unha-de-gato; urtiga	South America	Benzimento and prayer; ritual baths; protection and cleansing; relief of inflammation, anxiety, or agitation; domestic and community-based care.
Rubiaceae	9	4.2%	*Canthium* sp.; *Dimetia scandens* (Roxb.) R.J.Wang; *Morinda citrifolia* L.; *Paederia foetida* L.; *Rothmannia wittii* (Craib) Bremek.; Karamū; *Oldenlandia corymbosa* L.; *Psychotria* sp.; *Psychotria viridis* Ruiz & Pav.	Asia; South America; Africa	General medicinal preparations; digestive and inflammatory complaints; ritual or psychoactive preparations; spiritual mediation and visionary practices.
Apocynaceae	8	3.8%	*Calotropis procera* (Aiton) W.T.Aiton; *Cionura erecta* (L.) Griseb.; *Holarrhena pubescens* Wall. ex G.Don; *Rauvolfia serpentina* (L.) Benth. ex Kurz; *Rhazya stricta* Decne.; *Cascabela thevetia* (L.) Lippold; *Apteranthes tuberculata* (N.E.Br.) Meve & Liede; *Periploca aphylla* Decne.	Asia; Africa	Digestive, inflammatory, cardiovascular, and topical complaints; oral medicinal preparations; general therapeutic uses documented by traditional healers.
Lycopodiaceae	8	3.8%	*Huperzia brevifolia* (Grev. & Hook.) Holub; *Huperzia columnaris* B.Øllg.; *Huperzia compacta* (Rothm.) Holub; *Huperzia crassa* (Humb. & Bonpl. ex Willd.) Rothm.; *Huperzia espinosana* B.Øllg.; *Huperzia tetragona* (Hook. & Grev.) Trevis.; *Huperzia weberbaueri* B.Øllg.; Lycopodiaceae spp.	South America	Sacred and psychoactive uses; spiritual mediation; ritual cleansing; diagnosis, divination, or visionary practices; treatment of culturally defined afflictions.
Rutaceae	7	3.3%	*Aegle marmelos* (L.) Corrêa; *Citrus* × *aurantiifolia* (Christm.) Swingle; *Citrus* × *aurantium* L.; *Citrus × limon* (L.) Osbeck; *Citrus* spp.; *Ruta graveolens* L.; *Ruta* spp.	Asia; South America	Teas and infusions; digestive complaints; aromatic baths and washes; protection, spiritual cleansing, and other ritual applications.
Poaceae	6	2.8%	*Cymbopogon citratus* (DC.) Stapf; *Cymbopogon densiflorus* (Steud.) Stapf; *Oryza sativa* L.; Poaceae spp.; *Saccharum officinarum* L.; *Setaria italica* (L.) P.Beauv.	Asia; South America	Teas and decoctions; digestive and febrile complaints; baths and cleansing; food-related preparations; offerings and other forms of ritual material support.

Representative uses summarize documentary associations reported for one or more botanical records within each family or identification group and should not be interpreted as properties shared by all taxa listed in the corresponding row. Use categories were non-exclusive, and individual plants could be associated with more than one therapeutic, ritual, spiritual, or community-based application. Complete plant-level associations, including specialists, geographical contexts, specific uses, taxonomic resolution, documentary qualifications, and bibliographic sources, are provided in Supplementary Table S14.

**Table 3:** Non-exclusive plant-use categories recoded from botanical records.

Recoded Use Category	Records	Percentage	Specific Documented Applications	Representative Plant Taxa	Interpretive Note
Prayer, benzimento, and performative speech	86	40.6%	Blessing and prayer rituals; opening paths; treatment of quebrante, embruxado, erysipelas, infections, inflammation, and culturally defined afflictions; energetic cleansing conducted through prayer.	*Justicia gendarussa* Burm.f.; *Alternanthera brasiliana* (L.) Kuntze; *Mangifera indica* L.; *Coriandrum sativum* L.; *Petiveria alliacea* L.; *Ruta graveolens* L.	Plants are activated through prayer, blessing, intention, gesture, and ritually authorized speech rather than functioning only as isolated therapeutic substances.
Ingestion, tea, infusion, or decoction	79	37.3%	Teas and oral preparations for infections, erysipelas, rheumatism, stomachache, jaundice, digestive complaints, and bodily cleansing or purgation.	*Alternanthera brasiliana* (L.) Kuntze; *Foeniculum vulgare* Mill.; *Pimpinella anisum* L.; *Aristolochia triangularis* Cham.; *Bidens pilosa* L.; *Mentha* sp.	Connects ritual and community-healing contexts with simple pharmacotechnical procedures and oral administration, either independently or as part of broader therapeutic sequences.
Ritual, religious, or spiritual use	75	35.4%	Magical-religious ceremonies; treatment of physical, mental, emotional, and supernatural afflictions; sacred preparations; spiritual mediation; healing rituals; ritual protection and restoration of balance.	*Echinopsis pachanoi* (Britton & Rose) Friedrich & G.D.Rowley; *Brugmansia aurea* Lagerh.; *Brugmansia sanguinea* (Ruiz & Pav.) D.Don; *Brugmansia candida* Pers.; *Brugmansia suaveolens* (Humb. & Bonpl. ex Willd.) Bercht. & J.Presl; *Nicotiana rustica* L.; *Nicotiana tabacum* L.	Includes sacred plants, religiously or cosmologically marked uses, spiritual mediation, and plants incorporated into culturally situated ritual sequences.
Pain, fever, inflammation, and infection	67	31.6%	Infections, erysipelas, inflammation, rheumatism, fever, pain, contusions, and inflammatory manifestations associated with wounds or musculoskeletal injuries.	*Alternanthera brasiliana* (L.) Kuntze; *Coriandrum sativum* L.; *Foeniculum vulgare* Mill.; *Pimpinella anisum* L.; *Ochna rhizomatosa* Tiegh.; *Ochna schweinfurthiana* F.Hoffm.	Establishes a bridge between local therapeutic categories and approximate biomedical groupings without reducing culturally situated uses to biomedical diagnoses.
Digestive, hepatic, and jaundice	63	29.7%	Jaundice, “amarelão”, diarrhea, stomachache, hepatic complaints, digestive disorders, and purgative preparations intended to cleanse the organism.	*Boerhavia diffusa* L.; *Tinospora cordifolia* (Willd.) Hook.f. & Thomson; *Bidens pilosa* L.; *Mentha* sp.; *Echinopsis pachanoi* (Britton & Rose) Friedrich & G.D.Rowley.	Particularly relevant in Asian and Andean records, where oral preparations, purgation, jaundice treatment, and digestive complaints were frequently documented.
Protection, cleansing, and descarrego	41	19.3%	Opening paths; energetic cleansing; protection from negative energies, witchcraft, spiritual attack, quebrante, embruxado, mal aire, and susto; domestic and family protection.	*Justicia gendarussa* Burm.f.; *Mangifera indica* L.; *Dracaena trifasciata* (Prain) Mabb. [syn. *Sansevieria trifasciata* Prain]; *Petiveria alliacea* L.; *Ruta graveolens* L.; *Brugmansia* spp.; *Echinopsis pachanoi* (Britton & Rose) Friedrich & G.D.Rowley.	Central to culturally defined forms of vulnerability, spiritual aggression, purification, protection, and the restoration of relational balance.
Bones, fractures, and sprains	14	6.6%	Fractures, sprains, contusions, bone strengthening, reduction in inflammation and pain, and support for the healing of musculoskeletal injuries.	*Solidago chilensis* Meyen; *Ochna rhizomatosa* Tiegh.; *Ochna schweinfurthiana* F.Hoffm.; *Chasmanthera dependens* Hochst.; *Piliostigma thonningii* (Schumach.) Milne-Redh.; *Combretum sericeum* G.Don.	Represents specialized ethnomedicinal knowledge involving plant preparations, bodily application, immobilization practices, and the treatment of traumatic injuries.
Topical, bodily, or wound application	12	5.7%	Wounds, external injuries, inflammatory conditions, poultices, washes, pastes, compresses, and other preparations applied directly to the body.	*Ochna rhizomatosa* Tiegh.; *Ochna schweinfurthiana* F.Hoffm.; *Chasmanthera dependens* Hochst.; *Piliostigma thonningii* (Schumach.) Milne-Redh.; *Combretum sericeum* G.Don.	Includes applications in which direct contact among plant preparations, the affected body part, and therapeutic handling constitutes a central element of care.
Childbirth, puerperium, reproduction, and gynecology	12	5.7%	Female genital care; fertility disorders; prenatal and postnatal care; stimulation of breast-milk production; maternal hygiene; reproductive complaints; and care of babies and children.	*Piper betle* L.	Relates plant knowledge to midwives, traditional healers, women’s health, reproductive therapies, maternal care, and the continuity of community-based health practices.
Baths, washes, and hydrotherapeutic uses	10	4.7%	Ritual and medicinal baths for embruxado, quebrante, inflammation, erysipelas, contusions, spiritual cleansing, general blessing, and protection.	*Coriandrum sativum* L.; *Bidens pilosa* L.; *Solidago chilensis* Meyen; *Dracaena trifasciata* (Prain) Mabb.; *Lavandula* sp.; *Leonotis nepetifolia* (L.) R.Br.; *Salvia rosmarinus* L.; *Petiveria alliacea* L.; *Ruta graveolens* L.	Expresses the role of water as a material, sensory, therapeutic, and ritual mediator capable of articulating plants, bodily experience, prayer, and cleansing.
Diagnosis, divination, vision, or oracle	8	3.8%	Visionary diagnosis; induction of altered states of consciousness to recognize the patient’s condition; identification of supernatural afflictions; divination; and determination of appropriate treatment.	*Echinopsis pachanoi* (Britton & Rose) Friedrich & G.D.Rowley; *Brugmansia aurea* Lagerh.; *Brugmansia sanguinea* (Ruiz & Pav.) D.Don; *Brugmansia candida* Pers.; *Brugmansia suaveolens* (Humb. & Bonpl. ex Willd.) Bercht. & J.Presl; *Nicotiana* spp.	Includes plants whose sensory or psychoactive properties are incorporated into culturally situated modes of seeing, knowing, diagnosing, and mediating with spiritual agencies.
Mental suffering, fear, fright, or nerves	8	3.8%	Calming uses; susto; fright; emotional distress; nervous-system alterations; fear; agitation; emotional release; and spiritual restoration.	*Salvia rosmarinus* L.; *Melissa officinalis* L.; *Tanacetum parthenium* (L.) Sch.Bip.; *Lobelia* sp.; *Echinopsis pachanoi* (Britton & Rose) Friedrich & G.D.Rowley.	Connects psychosocial suffering, culturally defined vulnerability, sensory experience, nervous complaints, and ritual or spiritual care.
Smoke, fumigation, tobacco, and inhalation	2	0.9%	Smoking, inhalation, nasal aspiration, ritual blowing, and administration of tobacco powder or liquor to produce stimulant, sedative, psychoactive, protective, or cleansing effects.	*Nicotiana rustica* L.; *Nicotiana tabacum* L.	Although documentarily underrepresented, this category demonstrates the importance of smoke, breath, aroma, and inhalation as material and performative mediators of healing.

Use categories were non-exclusive, and a single botanical entry could be assigned to more than one category when multiple applications were directly documented. Percentages were calculated using the 212 botanical entries as the denominator and should therefore not be summed to 100%.

**Table 5:** Integrative model for ritual healing specialists as mediators of plant-based community knowledge.

Analytical Layer	Components	Guiding Question	Interpretive Contribution
Botanical material	Plants, families, species, ethnospecies, parts, preparations, water, smoke, oils, baths, and objects.	Which materials are used, how are they prepared, and how reliable is the botanical evidence?	Makes visible the sensory, ecological, and potentially pharmacological basis of care.
Performative	Prayer, blessing, chant, whisper, sign, touch, blowing, hands, and ritual sequence.	How is healing enacted through word, body, and ritual performance?	Explains how healing is performed, and not merely prescribed.
Relational-cosmological	Saints, spirits, ancestors, divinities, territory, moral order, and non-human agencies.	Which agencies are considered active in the therapeutic process?	Avoids reduction to pharmacology or isolated belief.
Community-political	Legitimacy, gender, lineage, secrecy, memory, trust, legal status, religious discrimination, institutional inclusion or exclusion, interface with public health, and epistemic justice.	Who is authorized to heal, how is knowledge transmitted and protected, and which legal or institutional conditions enable or constrain the practice?	Connects ritual healing to cultural heritage, pluralism, rights, and biocultural conservation.

## Data Availability

All data supporting the descriptive analyses are provided in the Supplementary Materials, including the formal PRISMA-ScR pathway, the separately identified contextual supplementary records, and Supplementary Table S14 containing the detailed botanical matrix. No new interviews, field collections, or primary community data were generated for this review.

## Supplementary Materials

The following supporting information can be downloaded at https://www.mdpi.com/article/10.3390/plants15162467/s1: Table S1: Consistency check between the written results and the consolidated matrices; Table S2: Operational PRISMA-ScR pathway and corpus construction; Table S3: Most frequent harmonized specialist terms in the 186-record corpus; Table S4: Ritual dimensions coded in the analytical records; Table S5: Distribution of plant records by continental attribution; Table S6: Main descriptive indicators of the analyzed plant matrix; Figure S1: Top 15 botanical families by species richness; Figure S2: Top 15 countries or canonical regions by plant-species richness; Table S7: Harmonized specialist terms associated with plant records; Table S8: Article records with the largest contributions to the plant matrix; Figure S3: Article codes with the largest number of linked plant records; Table S9: Examples of plant records with multiple documentary or continental links; Table S10: Recoded non-exclusive plant-use categories retained in the consolidated file; Figure S4: Use categories ranked by plant-species richness; Figure S5: Original specialist denominations ranked by the number of linked plant records in the assembled documentary corpus. Values are not estimates of cultural prevalence or knowledge breadth; Figure S6: Descriptive alluvial map linking specialist category, botanical family, and plant-use category. Flow widths represent documentary frequencies in the assembled matrix; Figure S7: Descriptive alluvial map linking botanical family, continent, and canonical country or region. Flow widths represent documentary frequencies and are not standardized by flora size or sampling effort; Table S11: Descriptive synthesis of the principal documentary flows represented in Figures S6 and S7; Table S12: Descriptive species-record overlap among South America, Asia, and Africa in the assembled matrix; Table S13: Interpretive synthesis of the documentary patterns represented in the bipartite networks; Figure S8: Descriptive Venn diagram of species-record overlap among South America, Asia, and Africa in the assembled matrix. Counts are not standardized by regional flora or sampling effort; Figure S9: Descriptive bipartite networks of documentary links: (A) botanical families and canonical countries or regions; (B) specialist denominations and plant-use categories. Node sizes and edge weights represent frequencies in the assembled matrix; Figure S10: Treemap of documentary links among continent, country or region, and botanical family. Proportional areas represent the assembled corpus and not ecological abundance, cultural prevalence, or standardized regional richness; Figure S11: Documentary allocation of specialist records and botanical entries by continental grouping in the consolidated corpus. Blue bars count analytical source records assigned to each grouping; orange bars count plant-entry attributions extracted from those sources. The two series have different units and are not measures of cultural prevalence or floristic diversity; Figure S12: Co-documentation heat map linking original specialist denominations to non-exclusive plant-use categories. Rows preserve the denomination recorded in the source; columns are recoded use categories; each cell is the number of source-coded botanical entries linking the two fields. Cell values combine label frequency and botanical detail per source and are not estimates of prevalence or cultural importance; Table S14: Detailed list of the 212 botanical entries, source-cited and consolidated names, taxonomic resolution, vernacular or ethnospecies information when available, documented uses and applications, specialist associations, geographical context, documentary qualifications, and bibliographic record codes.

## Abbreviations

The following abbreviations are used in this manuscript:

AChE, acetylcholinesterase; CAPES, Coordenação de Aperfeiçoamento de Pessoal de Nível Superior; CC BY, Creative Commons Attribution; CT-Agro, Sectoral Fund for Agribusiness; DOI, Digital Object Identifier; FAPESB, Fundação de Amparo à Pesquisa do Estado da Bahia; FINEP, Financiadora de Estudos e Projetos; FNDCT, Fundo Nacional de Desenvolvimento Científico e Tecnológico; JBI, Joanna Briggs Institute; MAO, monoamine oxidase; MCTI, Ministério da Ciência, Tecnologia e Inovação; PCC, Population–Concept–Context; POWO, Plants of the World Online; PRISMA-ScR, Preferred Reporting Items for Systematic Reviews and Meta-Analyses Extension for Scoping Reviews; PRPPG, Pró-Reitoria de Pesquisa e Pós-Graduação; WCVP, World Checklist of Vascular Plants; WHO, World Health Organization.
