# Supplementary material for: Between Leaves, Prayers, and Healing Hands: A Cross-Regional Scoping Review of Ritual Healers and Plant-Based Community Knowledge
Source: Plants (Basel). 2026 Aug 14;15(16):2467. doi: 10.3390/plants15162467 (PMC13516167; doi:10.3390/plants15162467)
Supplement: Supplementary file 1 [file plants-15-02467-s001.zip › Supplementary Materials.pdf]

Supplementary Materials

S1. Corpus verification and analytical scope

S1.1. Consistency checks across analytical files and matrices

The apparent difference between the 212 plant records and the 225 continental plant attributions results from multiregional coding: a plant or record may contribute to more than one geographical grouping.

Table S1. Consistency check between the written results and the consolidated matrices.

Checked indicator	Value in consolidated text
Records in Artigos_analisados	186
Records derived from the scoping review	54
Records derived from qualified manual identification	132
New records integrated in this version	44
Unique harmonized specialist terms	74
Integrated plant records	212
Registered botanical families	68
Records with taxonomic uncertainty	11
Plant continental attributions	225
Most frequent family	Lamiaceae = 20
Most frequent harmonized term	Traditional healer = 46

S1.2. PRISMA-ScR pathway and corpus composition

The operational review pathway identified 255 initial records, removed eight duplicates and screened 247 unique records by title and abstract. Of these, 61 were referred to the operational full-text eligibility stage, and 54 were retained in the scoping review. Qualified manual integration added records that improved intercultural and terminological coverage, resulting in a consolidated analytical corpus of 186 records. Therefore, the 186 records should be described as a consolidated analytical corpus rather than as the homogeneous output of a single systematic search.

Table S2. Operational PRISMA-ScR pathway and corpus construction.

PRISMA or operational step	Value
Records identified	255
Duplicates removed	8
Unique records screened by title/abstract	247
Records excluded after title/abstract screening	194
Articles retained after title/abstract screening	61
Records assessed in the operational full-text stage	61
Articles excluded in the operational full-text stage	7
Articles included after operational full-text assessment	54
Scoping-review records	54
Records identified manually	132
New records integrated in this version	44

PRISMA or operational step	Value
Total analytical records	186

S1.3. Harmonized specialist terms and terminological salience

The harmonized specialist field included 74 unique terms. The most frequent category was Traditional healer, followed by Healer, Benzedor(a)/rezador(a), Yachak/yachakkuna and Curandero/curandera. This pattern indicates the coexistence of broad academic translation labels and culturally situated emic denominations.

Table S3. Most frequent harmonized specialist terms in the 186-record corpus.

Harmonized term	Records	Percentage of 186
Traditional healer	46	24.7%
Healer	16	8.6%
Benzedor(a)/rezador(a)	10	5.4%
Yachak/yachakkuna	8	4.3%
Curandero/curandera	6	3.2%
Vaidya	4	2.2%
Raizeiro/raizeira	4	2.2%
Asian shamanic/mediumistic specialist	4	2.2%
Yatiri/Kallawaya	4	2.2%
Hakim / hakim	3	1.6%
Tohunga/Kahuna	3	1.6%
Sangoma	2	1.1%
Albularyo	2	1.1%
Rootworker/conjure doctor	2	1.1%
Cunning man/woman	2	1.1%
Dukun	2	1.1%
Sobador/huesero	2	1.1%
Szeptucha/szeptuch	2	1.1%
Tahu'a	2	1.1%
Vegetalista/Ayahuasquero	2	1.1%

S1.4. Ritual architecture in the analytical records

Seven ritual dimensions were coded to capture the coexistence of word, gesture, material supports, entity mediation, protection, institutional religious links and oracular diagnosis. Material supports formed the most frequent high-presence dimension, while word/prayer, mediation with entities and oracular diagnosis showed substantial but heterogeneous distribution.

Table S4. Ritual dimensions coded in the 186 analytical records.

Ritual dimension	High (H)	Medium (M)	Low (L)	ND	Synthetic reading
Word / prayer	43	84	13	46	Recurrent dimension; high or medium presence in most coded cases.
Body gesture	28	61	97	0	Cross-cutting dimension, with variation according to descriptive density.
Material supports	111	0	0	75	Dominant material axis; high presence in all determined cases.

Ritual dimension	High (H)	Medium (M)	Low (L)	ND	Synthetic reading
Mediation with entities	56	45	44	41	Expressive but heterogeneous dimension across systems.
Protection / cleansing	34	59	58	35	Transversal dimension associated with variable documentation density.
Link to institutional religion	23	57	58	48	Variable dimension, often dependent on how each source describes religious affiliation.
Oracular diagnosis	32	84	70	0	Central in some systems and intermediate in 84 records.

S2. Plant matrix: distribution, taxonomic structure and document concentration

S2.1. Plant records by continental attribution

The final plant matrix included 212 entries associated with specialists, uses, countries or regions, continents, article codes and taxonomic observations. Continental counts are attribution-based because multiregional records may be counted in more than one geographical grouping.

Table S5. Distribution of plant records by continental attribution.

Continent/geographic grouping	Plant records by attribution	Percentage of 212
South America	102	48.1%
Asia	79	37.3%
Africa	33	15.6%
Oceania	5	2.4%
Europe	4	1.9%
Transregional	2	0.9%

S2.2. Descriptive indicators of the plant matrix

Table S6. Main descriptive indicators of the analyzed plant-species matrix.

Indicator	Result
Unique species	212
Botanical families	68
Genera	166
Distinct records/articles	44
Canonical countries/regions	24
Continents/geographic categories	6
Normalized specialist categories	14
Use categories	9
Normalized use records	2,572

S2.3. Taxonomic concentration by botanical family

The plant repertoire showed an asymmetric taxonomic distribution, with the 15 most represented families accounting for 135 species, or 63.7% of the total richness. Lamiaceae, Asteraceae, Solanaceae and Fabaceae formed the main taxonomic core, while the long tail of less frequent families preserved strong local specificity.

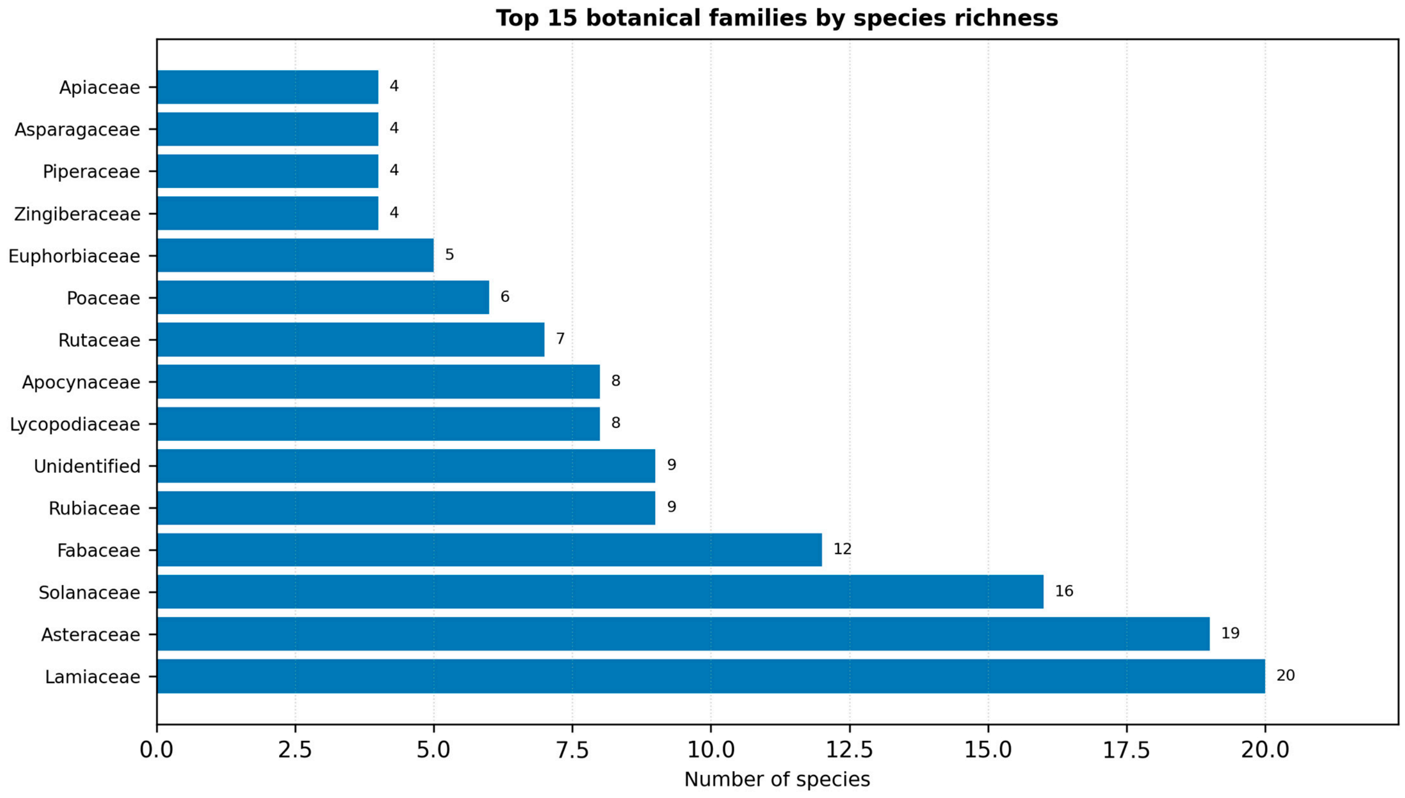

Figure S1. Top 15 botanical families by species richness.

S2.4. Geographic concentration by country or canonical region

The country-level pattern is source-driven rather than a standardized comparison of floristic or cultural richness. Brazil, India, and Ecuador supplied the largest numbers of botanical entries because the included studies from these settings contained comparatively extensive plant lists. In particular, record R0186 alone contributed 40 entries. Figure S2 therefore ranks documentary entries extracted from the included sources; it does not estimate the size or diversity of local healing repertoires.

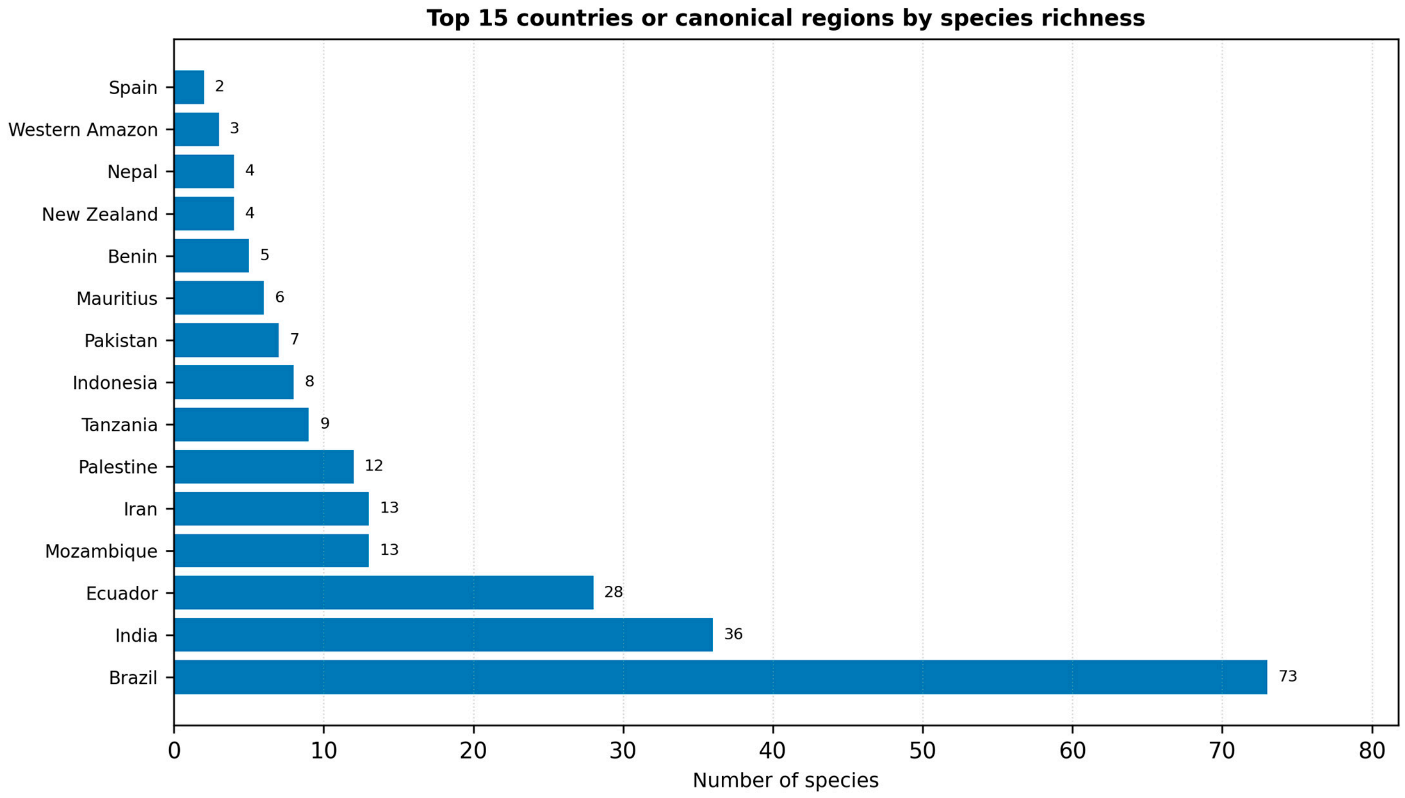

Figure S2. Top 15 countries or canonical regions by plant-species richness.

S2.5. Specialist terms associated with plant records

Linking botanical entries to article codes and then to harmonized specialist terms produced species–article attribution counts. “Traditional healer” accumulated 74 attributions, benzedor(a)/rezador(a) 65, “healer” 33, and yachak/yachakkuna 32. These values combine the number of sources assigned to a label with the number of extractable botanical entries in those sources and should be interpreted as source-structure indicators.

Table S7. Harmonized specialist terms associated with plant records.

Harmonized specialist term	Species-article attributions
Traditional healer	74
Benzedor(a)/rezador(a)	65
Healer	33
Yachak/yachakkuna	32
Raizeiro/raizeira	10
Hakim / hakim	8
Vaidya	7

Harmonized specialist term	Species-article attributions
Balian	6
Rongoa healer/tohunga	4
Meiga/meigo	2
Vegetalista/Ayahuasquero	2
Tahu'a	1
Guaritore/segnatore	1
Traditional health practitioner	1
Sikerei	1

Note: Counts are based on links between article code and plant record. A single plant may contribute to more than one attribution.

S2.6. Article-level contribution to botanical diversity

Botanical extraction was concentrated in a small number of sources. Bastos et al. (2024; R0186) contributed 40 of 212 entries (18.9%), Sharma et al. (2012; R0006) contributed 18 (8.5%), and record R0148 contributed 15 (7.1%). The five largest plant-list sources supplied 99 entries (46.7% of the matrix). Country, specialist, and family totals are therefore sensitive to article-level reporting depth and should be read together with Table S8 and Figure S3.

Table S8. Article records with the largest contributions to the plant matrix.

Code	Plant records	Short citation	Study topic	Country/region	Specialist
R0186	40	Bastos et al., 2024	Ethnobotany of blessing and plant-based healing in Chapada Diamantina National Park	Brazil, Bahia, Chapada Diamantina National Park, Andarai, Lencois and Mucuge	Benzedor(a)/rezador(a)
R0006	18	Sharma et al., 2012	Treatment of jaundice with medicinal plants in Indigenous communities of the Sub-Himalayan region	India	Traditional healer
R0148	15	Not specified in plant list	Plant-list reference associated with yachak/yachakkuna in Ecuador/Saraguro	Ecuador/Saraguro	Yachak/yachakkuna
R0014	13	Bruschi et al., 2011	Traditional healers and laypeople: qualitative and quantitative approach to local knowledge	Mozambique	Traditional healer
R0045	13	Hosseini et al., 2021	Ethnobotany of medicinal plants used by ethnic communities of Kerman province	Iran	Healer
R0043	12	Jaradat et al., 2016	Ethnopharmacological survey of medicinal plants used by herbalists and traditional healers	Palestine / West Bank	Traditional healer
R0180	11	Not specified in plant list	Plant-list reference associated with benzedeira/benzedor and prayer practices	Brazil, Santa Catarina	Benzedor(a)/rezador(a)
R0178	11	Not specified in plant list	Plant-list reference associated with benzedeira/benzedor in quilombola communities	Brazil, quilombos of Maranhao	Benzedor(a)/rezador(a)
R0003	10	P Sousa et al., 2021	Ethnoknowledge of medicinal and mystical plants used by healers in Juazeiro do Norte	Brazil	Healer
R0039	9	Panmei et al., 2019	Ethnobotany of medicinal plants used by the Zeliangrong ethnic group of Manipur	India	Healer
R0008	9	Peter et al., 2014	Ethno-medicinal knowledge and plants traditionally used to treat anemia in Tanzania	Tanzania	Traditional healer
R0152	7	Not specified in plant list	Plant-list reference associated with hakim in Pakistan/South Asia	Pakistan / South Asia	Hakim / hakim
R0010	7	Ojha et al., 2020	Ethnomedicinal knowledge of a marginal hill community of the Central Himalaya	India	Vaidya
R0001	7	Armijos et al., 2016	Phytochemical and ethnomedicinal study of Huperzia species used in traditional medicine	Ecuador	Yachak/yachakkuna
R0150	6	Not specified in plant list	Plant-list reference associated with raizeiro/raizeira in Campo Grande	Brazil, Campo Grande/MS	Raizeiro/raizeira

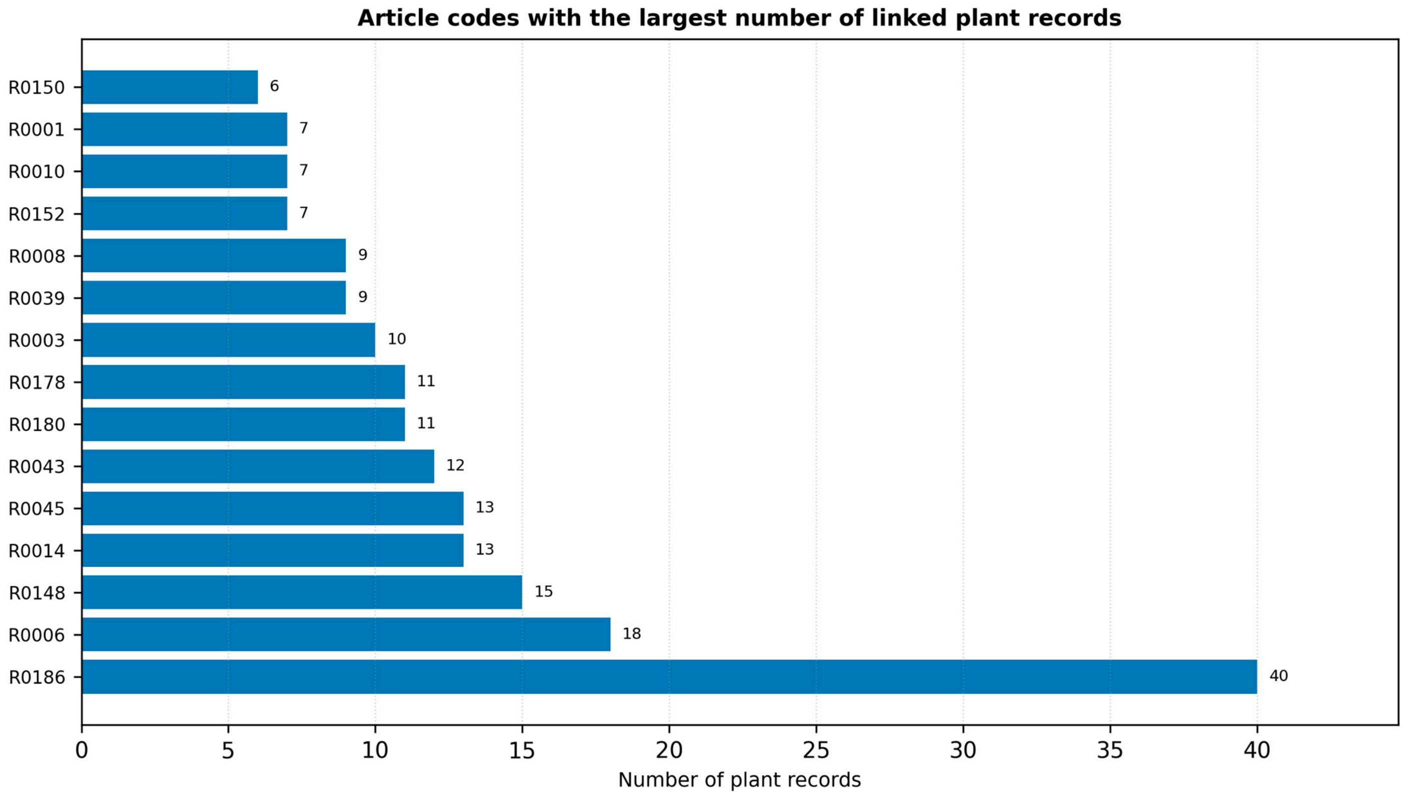

Figure S3. Article codes with the largest number of linked plant records.

S2.7. Multilinked plant records and analytical circulation of species

The plant matrix included records linked to more than one article code and/or more than one continental attribution. These cases indicate taxa that circulate across documentary, geographic and ritual contexts. Examples include Mangifera indica L., Holarrhena pubescens Wall. ex G.Don, Echinopsis pachanoi (Britton & Rose) H.Friedrich & G.D.Rowley and Rosmarinus officinalis L.

Table S9. Examples of plant records with multiple documentary or continental links.

Species or taxon	Number of linked codes	Codes	Continent(s)
Justicia gendarussa Burm.f.	2	R0180; R0186	South America
Achyranthes aspera L.	2	R0052; R0175	Asia
Alternanthera brasiliana (L.) Kuntze	2	R0180; R0186	South America
Mangifera indica L.	3	R0006; R0180; R0186	South America; Asia
Ozoroa reticulata (Baker f.) R.Fern. & A.Fern.	2	R0008; R0014	Africa
Holarrhena pubescens Wall. ex G.Don	2	R0006; R0014	Asia; Africa
Rauvolfia serpentina (L.) Benth. ex Kurz	2	R0036; R0052	Asia
Oroxylum indicum (L.) Kurz	2	R0006; R0039	Asia

Species or taxon	Number of linked codes	Codes	Continent(s)
Echinopsis pachanoi (Britton & Rose) H.Friedrich & G.D.Rowley	3	R0007; R0083; R0147	South America
Ricinus communis L.	2	R0006; R0186	Asia; South America
Jatropha gossypiifolia L.	2	R0178; R0186	South America
Ocimum basilicum L.	3	R0010; R0178; R0186	South America; Asia
Melissa officinalis L.	2	R0178; R0186	South America
Rosmarinus officinalis L.	4	R0149; R0181; R0184; R0186	South America; Europe
Lawsonia inermis L.	2	R0006; R0008	Asia; Africa

S2.8. Non-exclusive plant-use categories and pharmacobotanical-ritual overlap

Plant-use categories were coded as non-exclusive. Therefore, categories should not be summed as mutually exclusive classes. The same plant may be coded as a prayer resource, a bath material, a tea, a protection agent and a remedy for inflammation, jaundice, wounds or relational suffering. This overlap is an analytical finding because the corpus does not separate pharmacology, symbolism, spirituality and community care into rigid domains.

Table S10. Recoded non-exclusive plant-use categories retained in the consolidated file.

Recoded plant-use category	Records	Percentage of 212
Prayer, blessing and performative word	86	40.6%
Ingestion, tea, infusion or decoction	79	37.3%
Ritual, religious or spiritual use	75	35.4%
Pain, fever, inflammation and infection	67	31.6%
Digestive, hepatic and jaundice-related use	63	29.7%
Protection, cleansing and spiritual discharge	41	19.3%
Bones, fractures and sprains	14	6.6%
Topical, bodily or wound application	12	5.7%
Childbirth, postpartum, reproduction and gynecology	12	5.7%
Baths, washes and hydrotherapeutic uses	10	4.7%
Diagnosis, divination, vision or oracle	8	3.8%
Mental suffering, fear, susto or nerves	8	3.8%
Smoke, fumigation, smoking and inhalation	2	0.9%

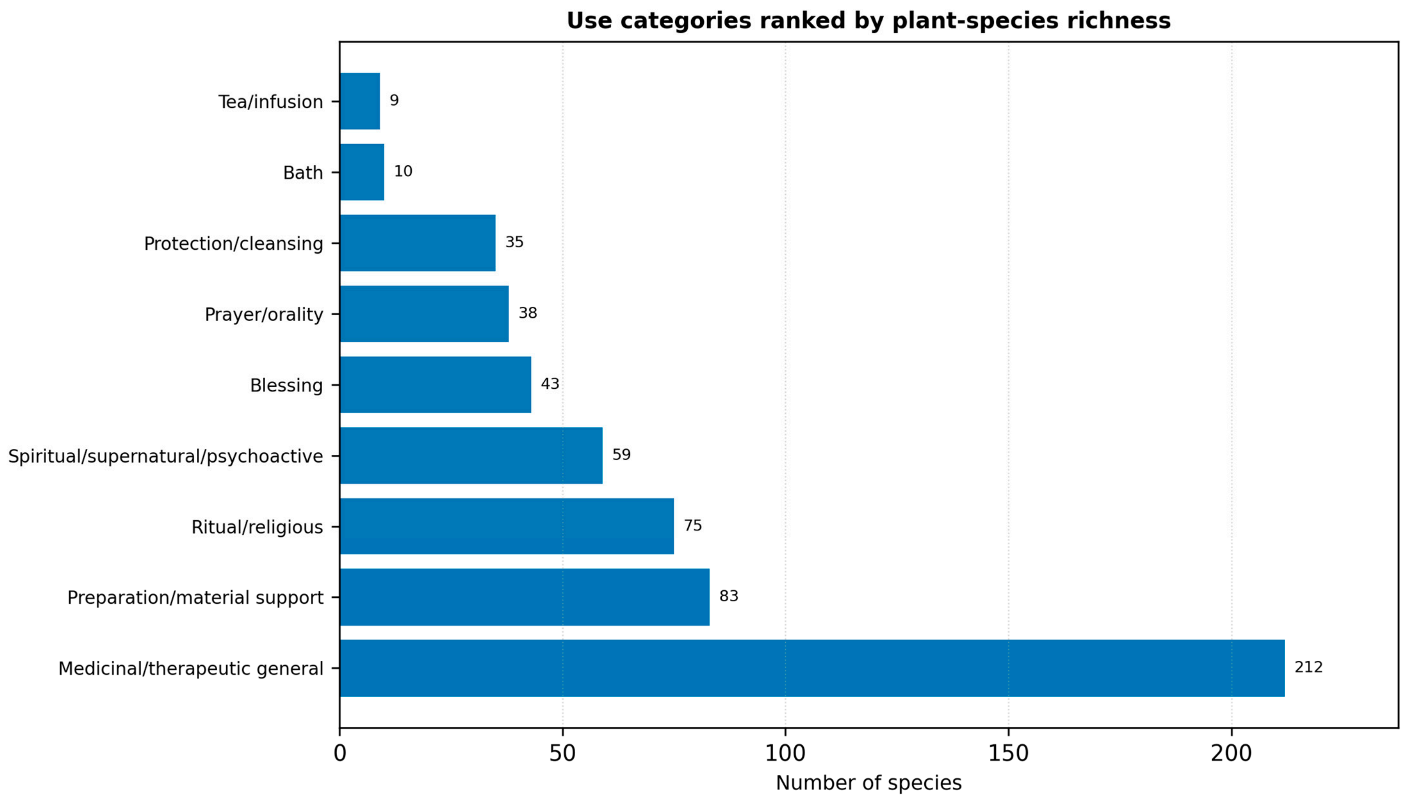

Figure S4. Use categories ranked by plant-species richness.

S3. Exploratory visualizations and interpretive safeguards

S3.1. Analytical units and reading rules for supplementary visualizations

Each visualization in this section answers a specific data-audit question. In Figure S5, bar length is the number of source-coded plant links associated with each original specialist denomination. In Figures S6 and S7, flow width is the number of plant-matrix rows connecting the displayed categories. In Figure S8, each set and overlap count is the number of unique taxon labels assigned to the indicated continental groupings. In Figure S9, an edge represents a documented co-attribution, edge weight is the number of linked rows, and node size summarizes incident links. In Figure S10, rectangle area is the number of plant records nested within continent, country or region, and botanical family. Supplementary Figures S11 and S12 preserve visualizations transferred from the main text; their units are defined in the accompanying captions and explanatory paragraphs.

These outputs describe the structure of the assembled documentary matrices. They are not normalized by the number of publications, communities, participants, historical periods, locally accessible species, or sampling effort, and they are not used for inferential tests or cross-regional rankings. Their purpose is to make the coding links auditable and to show how a small number of detailed sources can shape absolute frequencies.

S3.2. Documentary distribution by original specialist denomination

Original specialist denominations were retained to preserve ethnographic specificity. Figure S5 shows only the number of plant-record links retrieved for each denomination; it does not measure the breadth of local knowledge or the cultural centrality of any specialist group. Broad translated labels may accumulate more links because they are easier to index and may aggregate heterogeneous traditions.

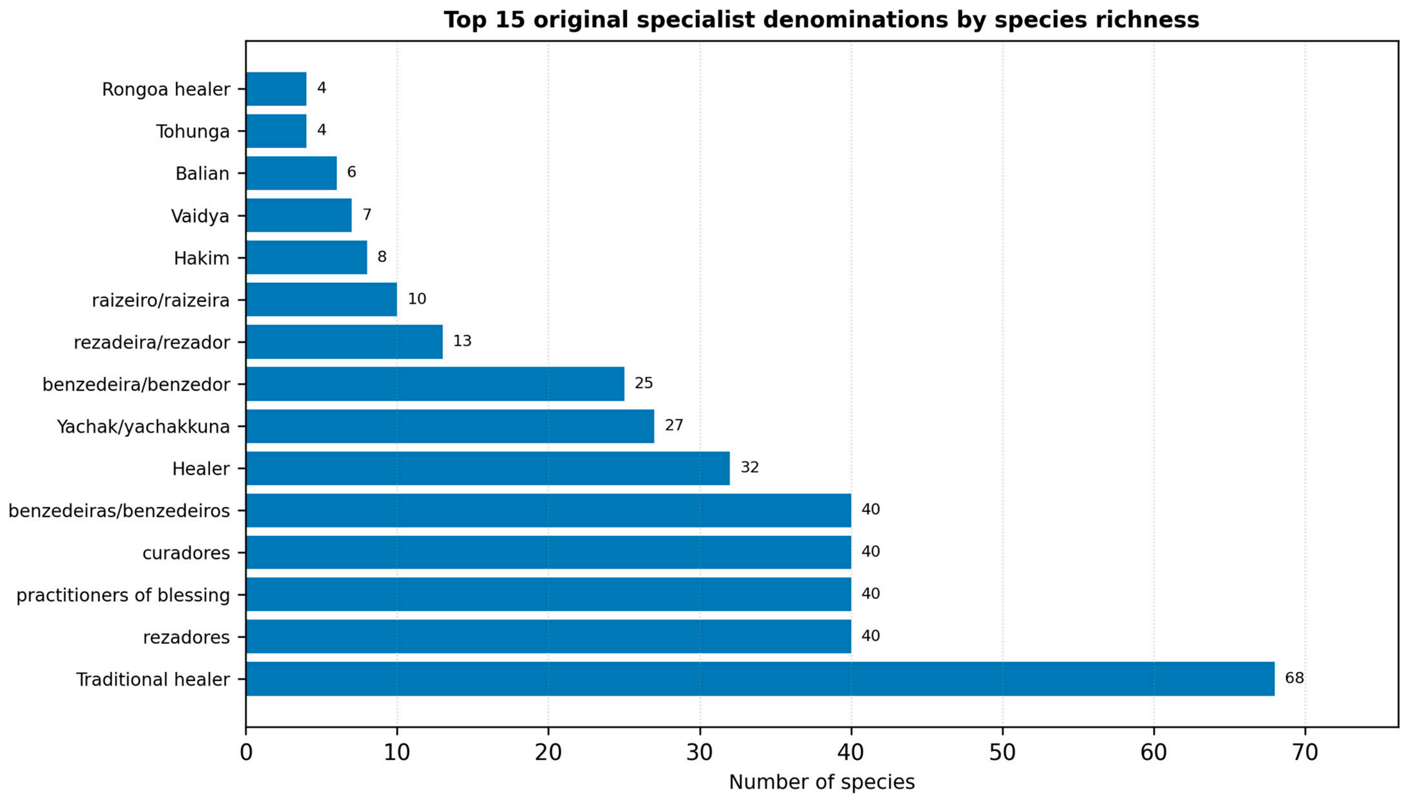

Figure S5. Original specialist denominations ranked by the number of linked plant records in the assembled documentary corpus. Values are not estimates of cultural prevalence or knowledge breadth.

S3.3. Documentary alluvial maps

Two alluvial diagrams separate the sociobotanical axis from the biogeographic axis. The first represents links from specialist category to botanical family and plant-use category; the second represents links from botanical family to continent and country or region. Flow widths are documentary frequencies derived from the normalized matrix and must not be interpreted as ecological abundance, cultural prevalence, historical continuity, or standardized regional richness.

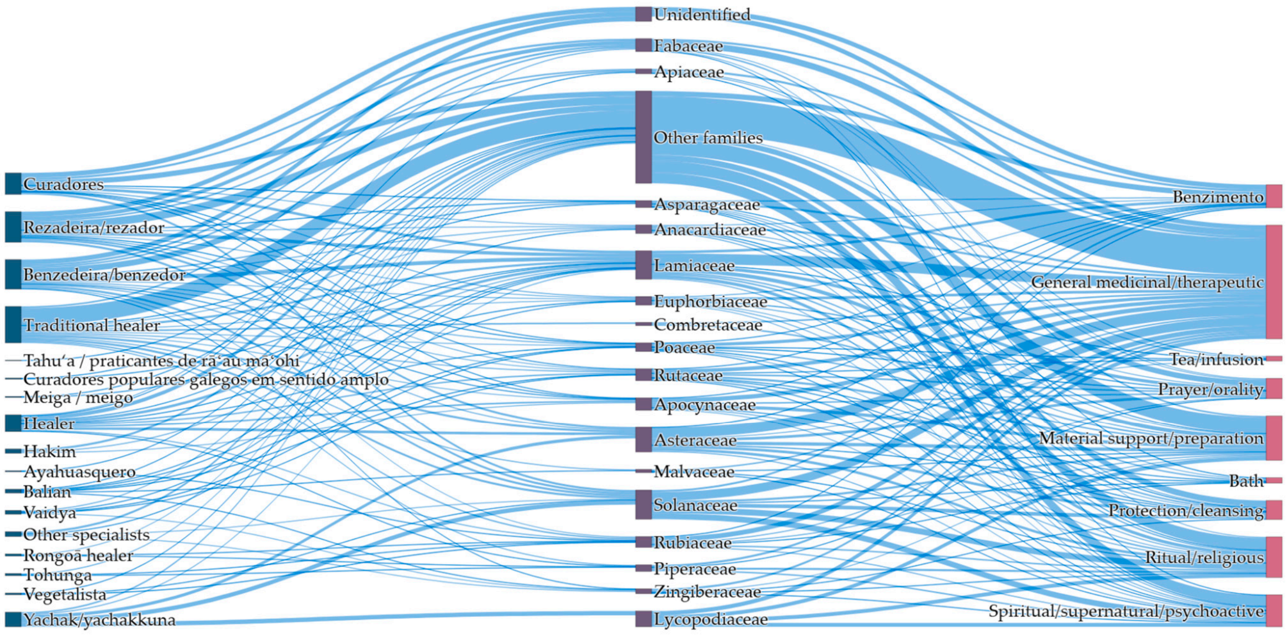

Figure S6. Descriptive alluvial map linking specialist category, botanical family, and plant-use category. Flow widths represent documentary frequencies in the assembled matrix.

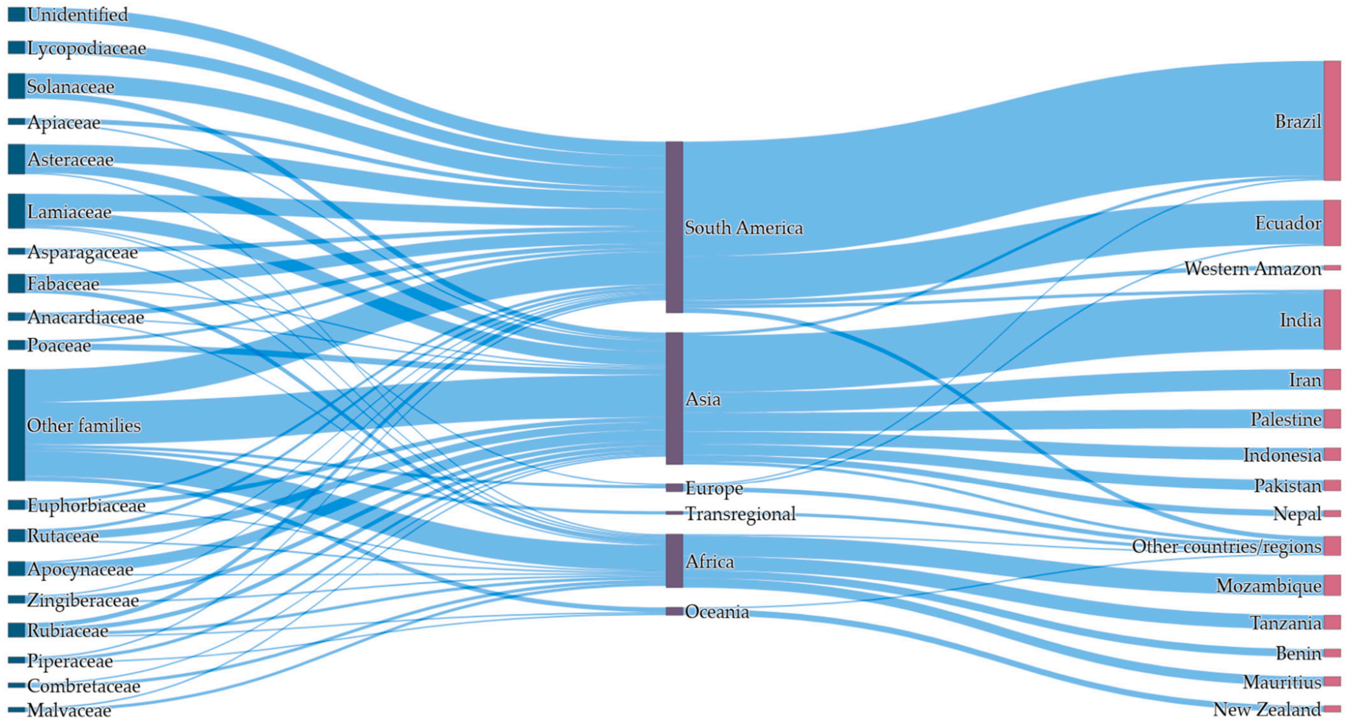

Figure S7. Descriptive alluvial map linking botanical family, continent, and canonical country or region. Flow widths represent documentary frequencies and are not standardized by flora size or sampling effort.

Table S11. Descriptive synthesis of the principal documentary flows represented in Figures S6 and S7.

Diagram	Highlighted flow	Value
Specialist-family-use	Other families -> Medicinal/therapeutic general	77
Specialist-family-use	Traditional healer -> Other families	39
Specialist-family-use	Lamiaceae -> Medicinal/therapeutic general	20
Specialist-family-use	Asteraceae -> Medicinal/therapeutic general	19
Specialist-family-use	Solanaceae -> Ritual/religious; Spiritual/supernatural/psychoactive	11 + 11
Family-continent-country	South America -> Brazil	73
Family-continent-country	Asia -> India	36
Family-continent-country	South America -> Ecuador	28
Family-continent-country	Other families -> Asia	29
Family-continent-country	Unidentified -> South America	9

S3.4. Supplementary species-sharing, network, and hierarchical visualizations

The Venn diagram, bipartite networks, and treemap are presented as descriptive supplements. Overlap, edge weight, and proportional area reflect the assembled matrix only. They were not standardized against regional flora size, habitat, plant-resource access, number of communities, historical period, or study effort and should not be interpreted as actual floristic similarity or territorial importance. The figures are retained to allow inspection of documentary structure while keeping the manuscript interpretation qualitative and limitation-centered.

Table S12. Descriptive species-record overlap among South America, Asia, and Africa in the assembled matrix.

Comparison	Species exclusive to the first set	Shared species	Species exclusive to the second set
South America vs Africa	101	0	33
South America vs Asia	96	5	74
Asia vs Africa	77	2	31
South America vs Asia vs Africa	96 / 77 / 31	0	5 shared between South America and Asia; 2 shared between Asia and Africa

Table S13. Interpretive synthesis of the documentary patterns represented in the bipartite networks.

Analyzed network	Main connectivity core	Peripheral pattern	Ethnobotanical interpretation
Original specialist x plants	traditional healer; benzedeiras/benzedeiros; curadores; rezadores	Many species linked to few specialist denominations; several local specialists with narrow repertoires.	Coexistence of shared plant repertories and locally specific ritual-care repertoires.
Original specialist x use category	Medicinal/therapeutic general; ritual/religious; spiritual/supernatural/psychoactive; blessing; protection/cleansing	Local specialists connected to fewer use categories.	Integration of therapeutic practice, ritual action, protection and oral performance.
Family x use category	Lamiaceae; Asteraceae; Solanaceae; Fabaceae; Other families	Long tail of families with low frequency and localized links.	A taxonomic core coexists with dispersed, culturally situated plant uses.

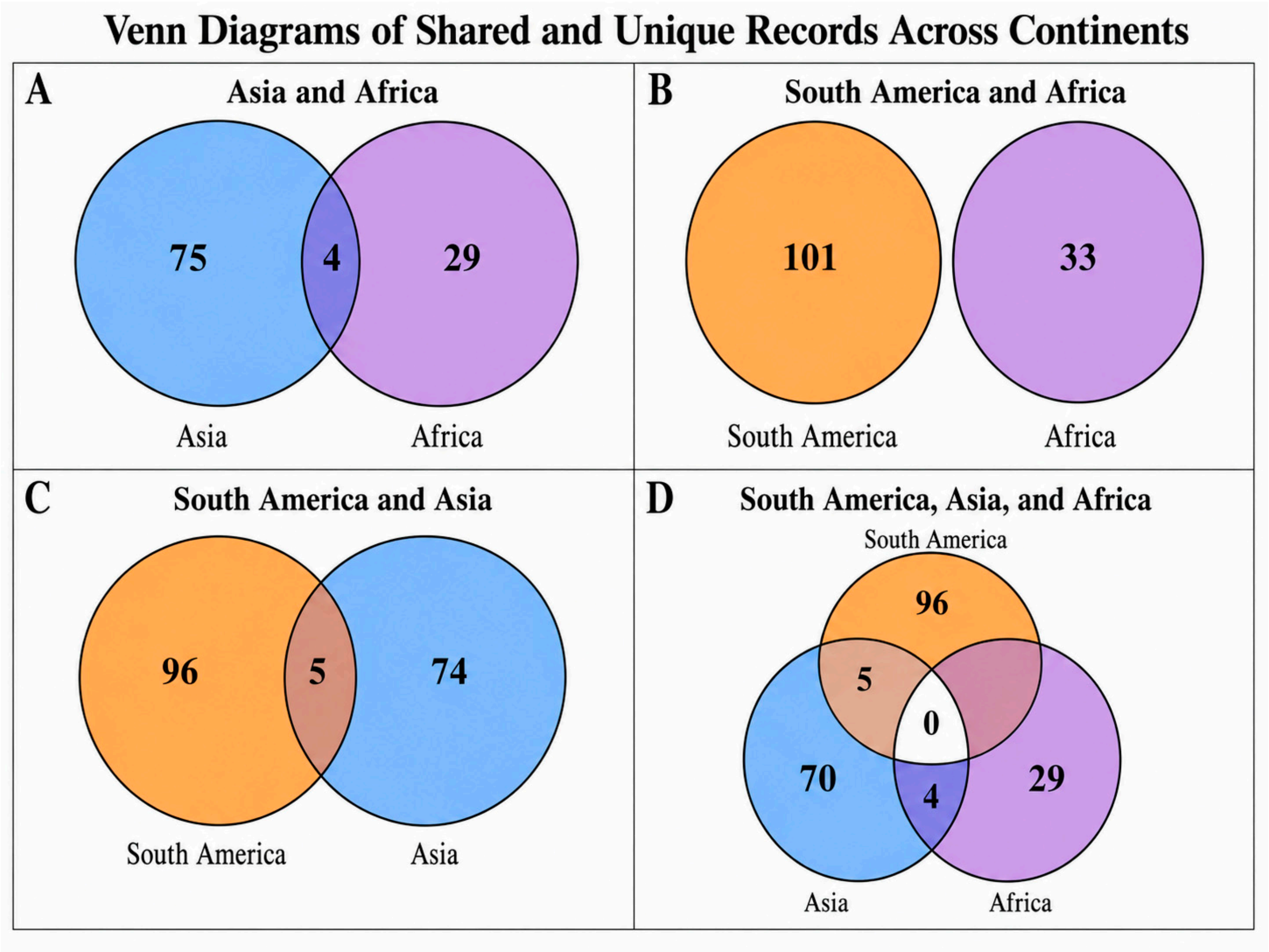

Figure S8. Descriptive Venn diagram of species-record overlap among South America, Asia, and Africa in the assembled matrix. Counts are not standardized by regional flora or sampling effort.

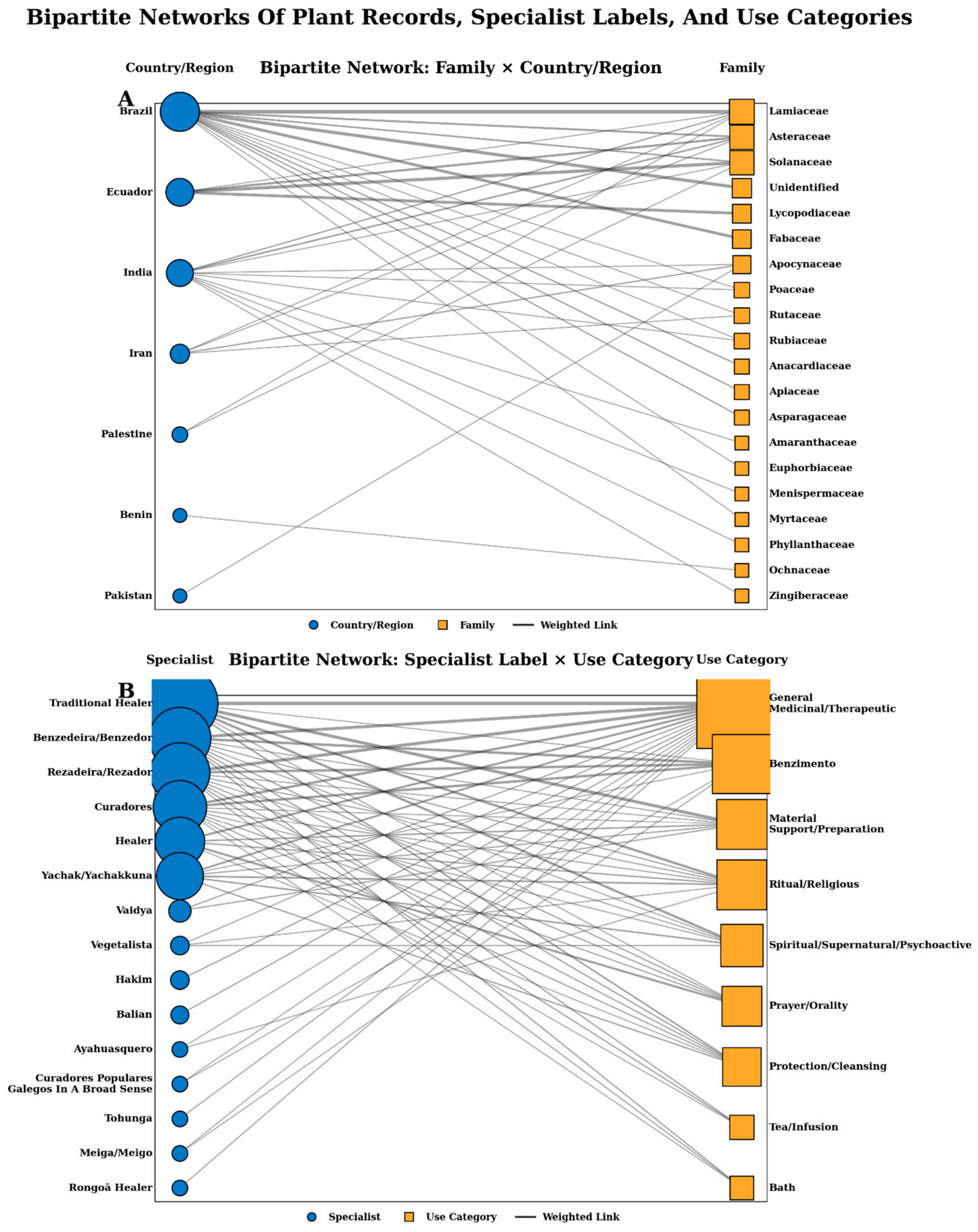

Figure S9. Descriptive bipartite networks of documentary links: (A) botanical families and canonical countries or regions; (B) specialist denominations and plant-use categories. Node sizes and edge weights represent frequencies in the assembled matrix.

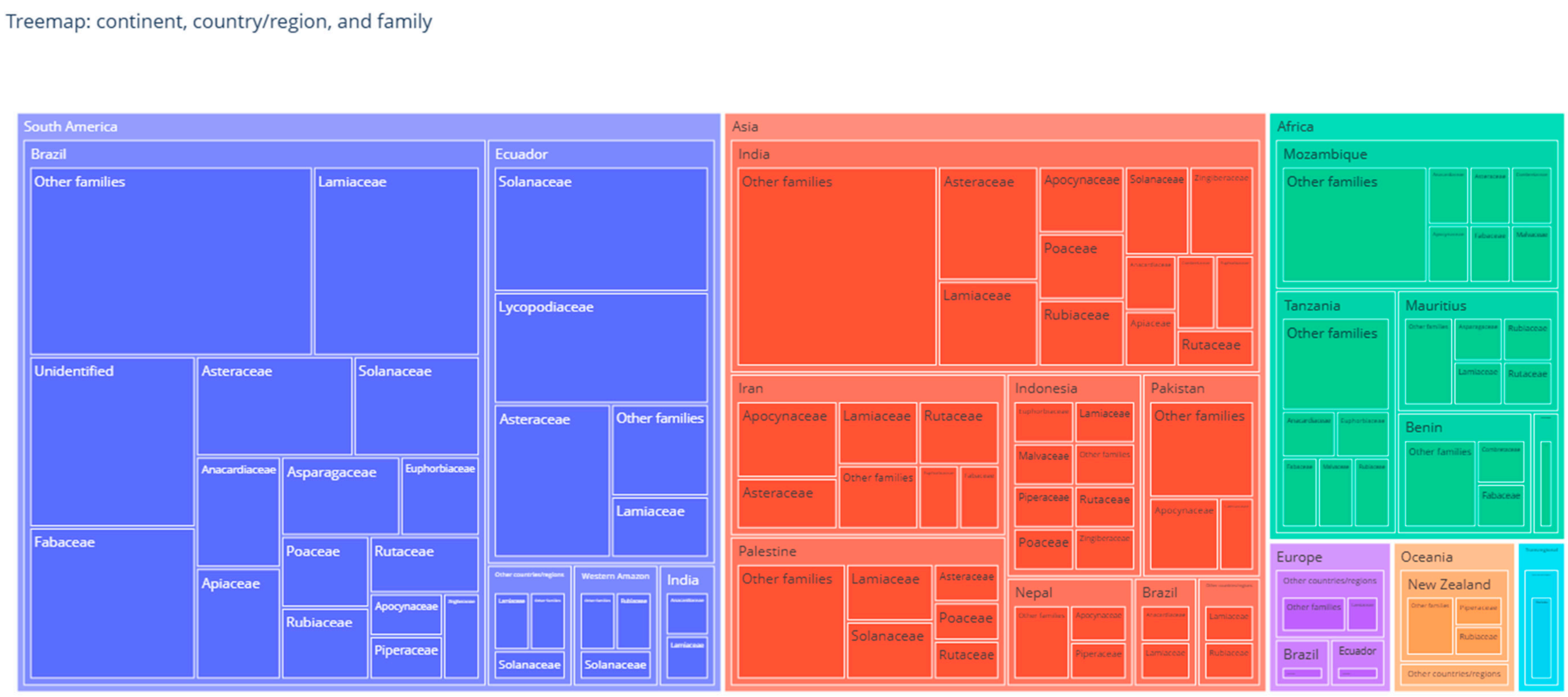

Figure S10. Treemap of documentary links among continent, country or region, and botanical family. Proportional areas represent the assembled corpus and not ecological abundance, cultural prevalence, or standardized regional richness.

S3.5. Visualizations transferred from the main text

The following displays were removed from the main text to prevent non-normalized absolute counts from carrying the visual weight of comparative results. They are retained here only for transparency, traceability of the revised manuscript, and inspection of the assembled data structure.

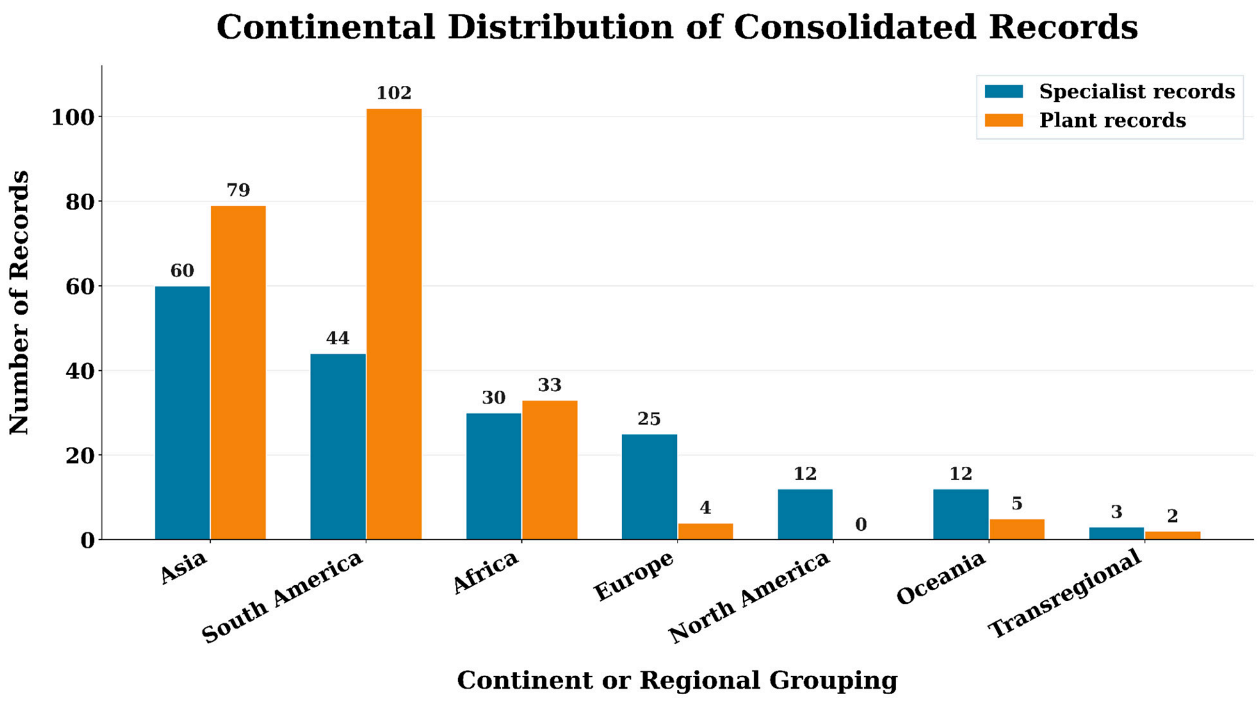

Figure S11. Documentary allocation of specialist records and botanical entries by continental grouping in the consolidated corpus. Blue bars count analytical source records assigned to each grouping; orange bars count plant-entry attributions extracted from those sources. The two series have different units and are not measures of cultural prevalence or floristic diversity.

Figure S11 shows extraction structure rather than a regional comparison. A source may contribute one specialist record but many botanical entries, while another may describe a specialist without an extractable plant list. The gap between the bars therefore reflects differences in source type and botanical reporting depth. It must not be interpreted as plant-to-specialist ratios, regional richness, or absence of plant-based practice.

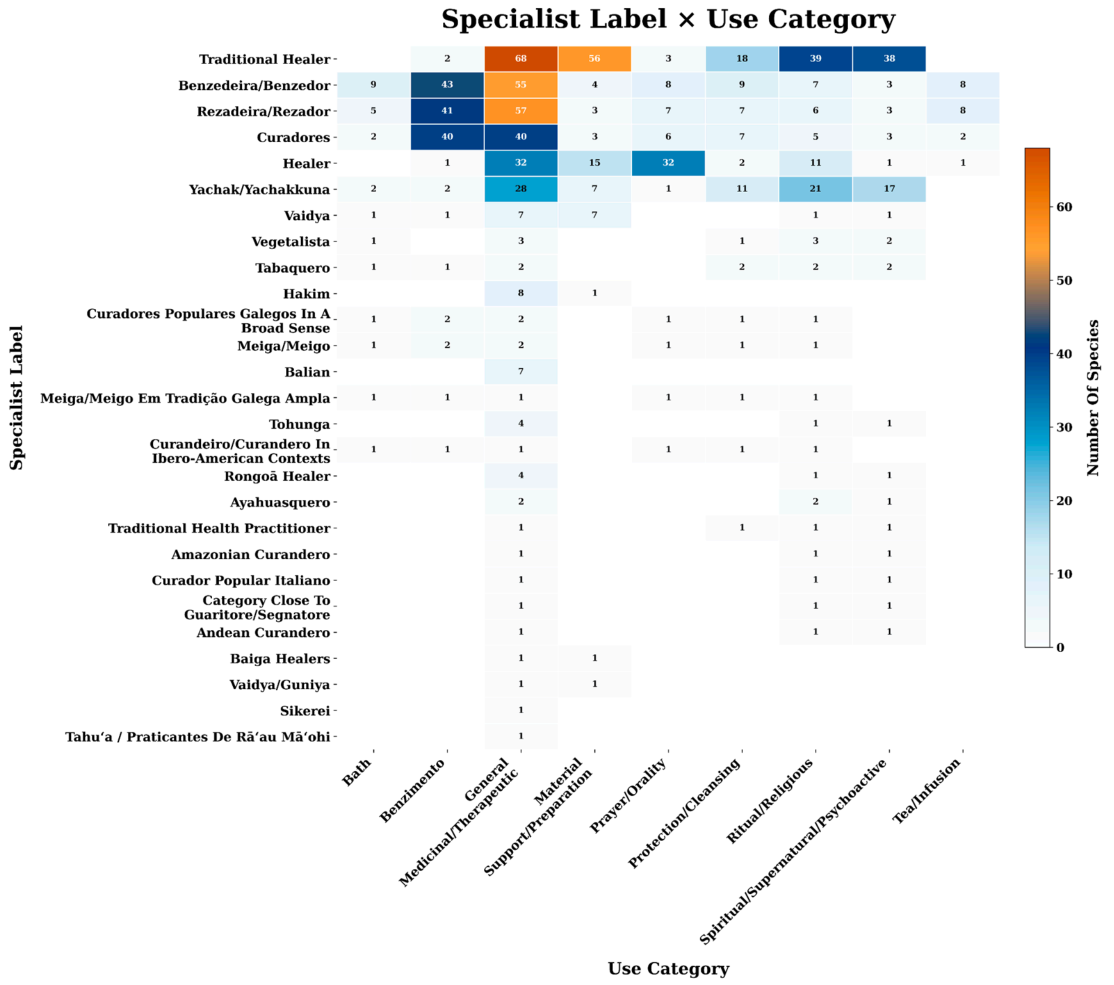

Figure S12. Co-documentation heat map linking original specialist denominations to non-exclusive plant-use categories. Rows preserve the denomination recorded in the source; columns are recoded use categories; each cell is the number of source-coded botanical entries linking the two fields. Cell values combine label frequency and botanical detail per source and are not estimates of prevalence or cultural importance.

The heat map answers only which labels and use categories were jointly documented in the assembled plant matrix. Broad translated labels may have more cells because they occur in more records, whereas specialist categories linked to a small number of plant-rich studies may also generate high counts. No cell represents the frequency of a practice among participants, co-use within a community, or equivalence among specialist traditions.
